# Supplementary material for: Metabolomics-Guided Discovery of New Dimeric Xanthones from Co-Cultures of Mangrove Endophytic Fungi Phomopsis asparagi DHS-48 and Phomopsis sp. DHS-11
Source: Mar Drugs. 2024 Feb 23;22(3):102. doi: 10.3390/md22030102 (PMC10971527; doi:10.3390/md22030102)
Supplement: Supplementary file 1 [file marinedrugs-22-00102-s001.zip › marinedrugs-2885840-supplementary.pdf]

Electronic Supplementary Information

Metabolomics-Guided Discovery of New Dimeric Xanthones from Co-cultures of Mangrove Endophytic Fungi *Phomopsis asparagi* DHS-48 and *Phomopsis* sp. DHS-11

Jingwan Wu, Dandan Chen, Qing Li, Ting Feng, Jing Xu*

Collaborative Innovation Center of Ecological Civilization, School of Chemistry and Chemical Engineering, Hainan University, Haikou 570228

* To whom correspondence should be addressed.

Prof. Dr. Jing Xu, Tel.: ++86–898–6627–9226, Fax: ++86–898–6627–9010, E–mail: *happyjing3@hainan.edu.cn*

Contents

Figure S1. ^{1}H-NMR of EtOAc extracts of *Phomopsis asparagi* DHS-48, *Phomopsis* sp. DHS-11 and their Co-culture measured in CD_3OD ---------- 4

Figure S2. PCA of metabolomics data of co-culture and their corresponding mono-cultures. 4

Figure S3. The MS/MS spectrum and possible fragmentation patterns of compound **5**-**9** and deacetylphomoxanthone B---------- 5

Figure S4. The MS/MS spectrum and possible fragmentation patterns of compound **1**-**4** ------ 6

Figure S5. ^{1}H-NMR of (**1**) ---------- 7

Figure S6. ^{13}C-NMR of (**1**)---------- 7

Figure S7. DEPT of (**1**) ---------- 8

Figure S8. ^{1}H-^{1}H COSY of (**1**) ---------- 8

Figure S9. HSQC of (**1**) ---------- 9

Figure S10. HMBC of (**1**) ---------- 9

Figure S11. NOSEY of (**1**) ----------10

Figure S12. HR-ESI-MS of (**1**) ----------10

Figure S13. ^{1}H-NMR of (**2**)----------11

Figure S14. ^{13}C-NMR of (**2**) ----------11

Figure S15. DEPT of (**2**)----------12

Figure S16. ^{1}H-^{1}H COSY of (**2**)----------12

Figure S17. HSQC of (**2**)----------13

Figure S18. HMBC of (**2**) ----------13

Figure S19. NOSEY of (**2**) ----------14

Figure S20. HR-ESI-MS of (**2**) ----------14

Figure S21. ^{1}H-NMR of (**3**)----------15

Figure S22. ^{13}C-NMR of (**3**) ----------15

Figure S23. DEPT of (**3**)----------16

Figure S24. ^{1}H-^{1}H COSY of (**3**)----------16

Figure S25. HSQC of (**3**)----------17

Figure S26. HMBC of (**3**) ----------17

Figure S27. NOSEY of (**3**) ----------18

Figure S28. HR-ESI-MS of (**3**) ----------18

Figure S29. ^{1}H-NMR of (**4**)----------19

Figure S30. ^{13}C-NMR of (**4**) ----------19

Figure S31. HR-ESI-MS of (**4**) ----------20

Figure S32. ^{1}H-NMR of (**5**)----------20

Figure S33. ^{13}C-NMR of (**5**) ----------21

Figure S34. HR-ESI-MS of (**5**) ---21
Figure S35. ^{1}H-NMR of (**6**)---22
Figure S36. ^{13}C-NMR of (**6**) ---22
Figure S37. HR-ESI-MS of (**6**) ---23
Figure S38. ^{1}H-NMR of (**7**)---23
Figure S39. ^{13}C-NMR of (**7**) ---24
Figure S40. HR-ESI-MS of (**7**) ---24
Figure S41. ^{1}H-NMR of (**8**)---25
Figure S42. ^{13}C-NMR of (**8**) ---25
Figure S43. HR-ESI-MS of (**8**) ---26
Figure S44. ^{1}H-NMR of (**9**)---26
Figure S45. ^{13}C-NMR of (**9**) ---27
Figure S46. HR-ESI-MS of (**9**) ---27
Figure S47. HPLC spectrum for the purity of tested compounds -----------------------------------27
Table S1. Putative annotation of metabolites produced in the non-cultures of *Phomopsis asparagi* DHS-48 and *Phomopsis* sp. DHS-11, and their co-culture. ---------------------------------- 32
Table S2. Gibbs free energies[a] and equilibrium populations[b] of low-energy conformers of phomoxanthone L (**1**)-- 37
Table S3. Cartesian coordinates for the low-energy reoptimized MMFF conformers of phomoxanthone L (**1**) at B3LYP/6-31G(d,p) level of theory in gas------------------------------------37
Table S4. Gibbs free energies[a] and equilibrium populations[b] of low-energy conformers of phomoxanthone M (**2**)--- 45
Table S5. Cartesian coordinates for the low-energy reoptimized MMFF conformers of phomoxanthone M (**2**) at B3LYP/6-31G(d,p) level of theory in gas-----------------------------------45
Table S6. Gibbs free energies[a] and equilibrium populations[b] of low-energy conformers of phomoxanthone N (**3**)-- 52
Table S7. Cartesian coordinates for the low-energy reoptimized MMFF conformers of phomoxanthone N (**3**) at B3LYP/6-31G(d,p) level of theory in gas-----------------------------------51

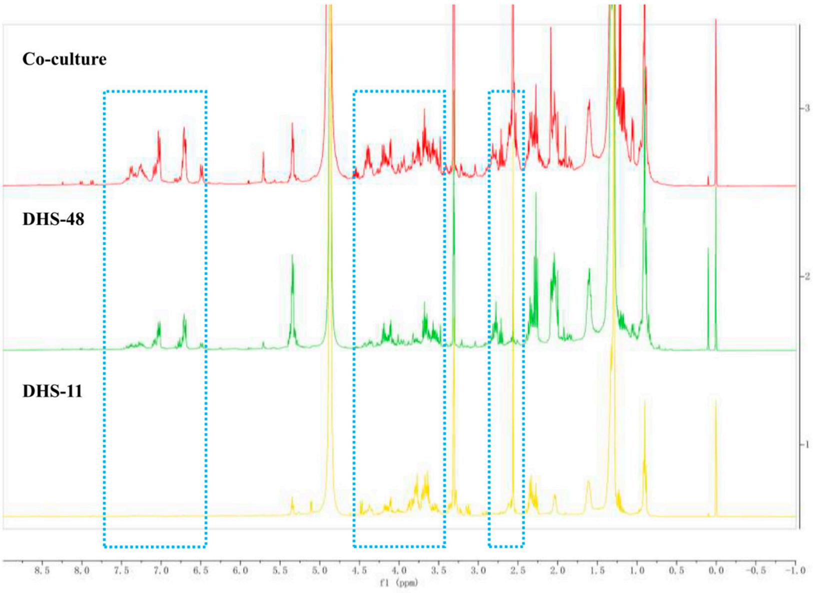

Figure S1. ^{1}H NMR spectra of EtOAc extracts of *Phomopsis asparagi* DHS-48, *Phomopsis* sp. DHS-11 and their Co-culture measured in CD_3OD at 400 MHz , chemical shifts (δ) presented in ppm.

Figure S2. PCA of metabolomics data of co-culture and their corresponding mono-cultures, (**A**) the score plots and (**B**) the loading plots. The data analyzed by LC-IT-TOF-MS in the positive mode (612 features). The parameters (R^2 and Q^2) of the score plots demonstrated the discriminative ability of this model. The scattered dots labeled with *m/z* in **B** were representative features mentioned in the results section.

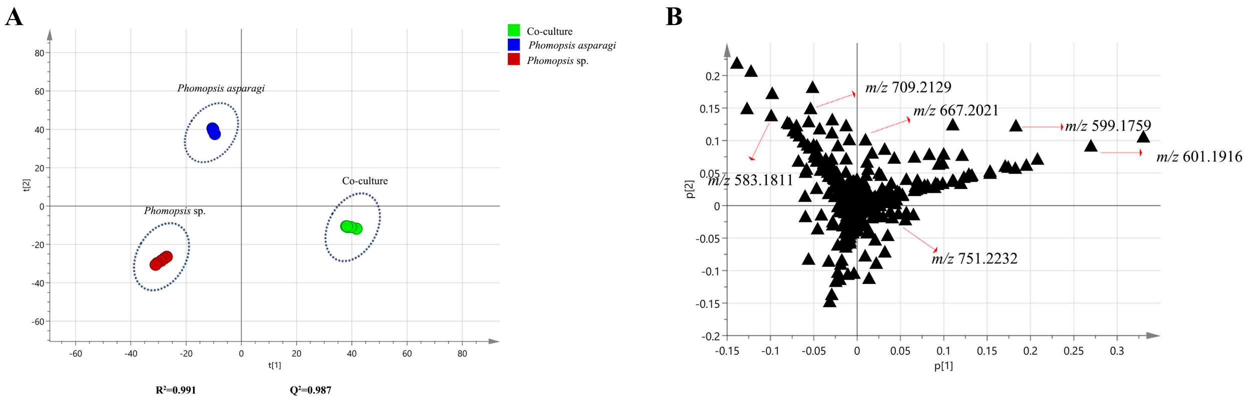

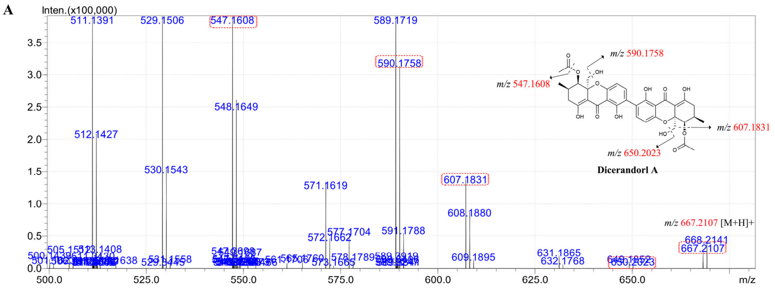

The (+)- MS/MS spectrum of compound **7** by the precursor of *m/z* 667.2107

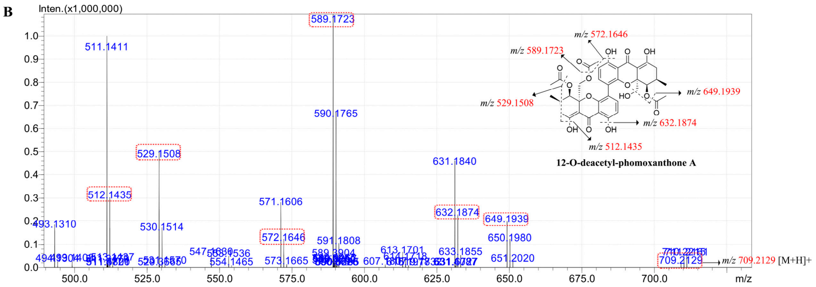

The (+)- MS/MS spectrum of compound **9** by the precursor of *m/z* 709.2129

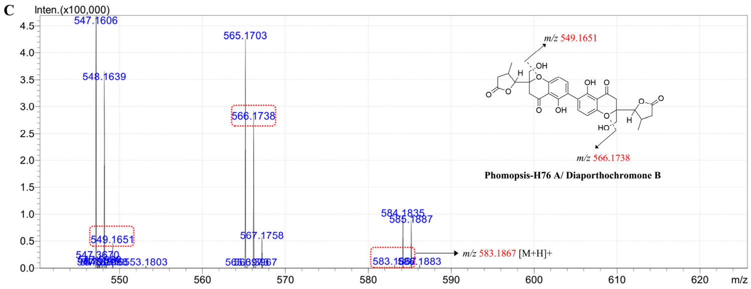

The (+)- MS/MS spectrum of compound **5**/**6** by the precursor of *m/z* 583.1867

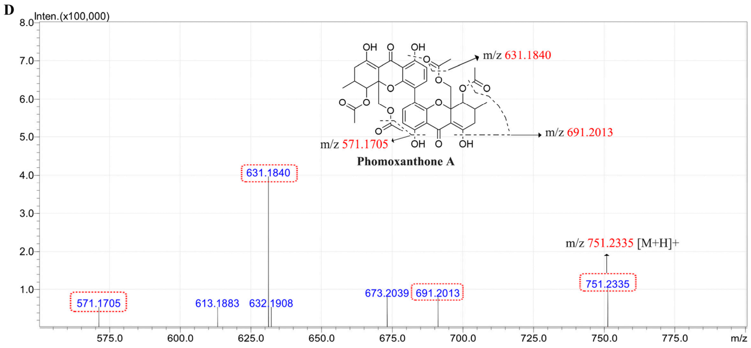

The (+)- MS/MS spectrum of compound **8** by the precursor of *m/z* 751.2335

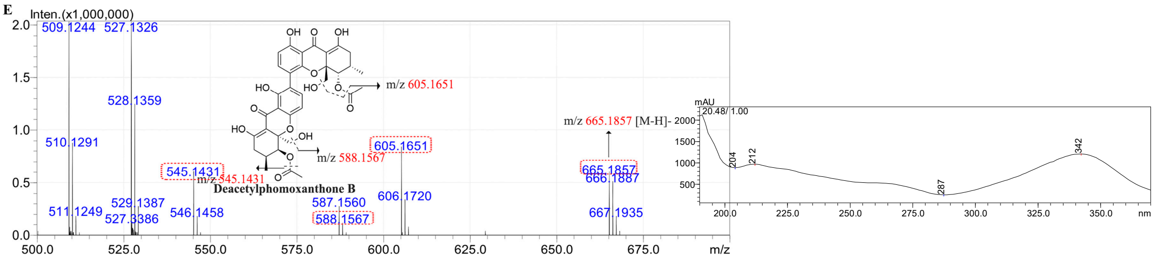

The (+)- MS/MS spectrum of compound **deacetylphomoxanthone B** by the precursor of *m/z* 665.1857

Figure S3. The MS/MS spectrum and possible fragmentation patterns of compound **5-9** and deacetylphomoxanthone B

A

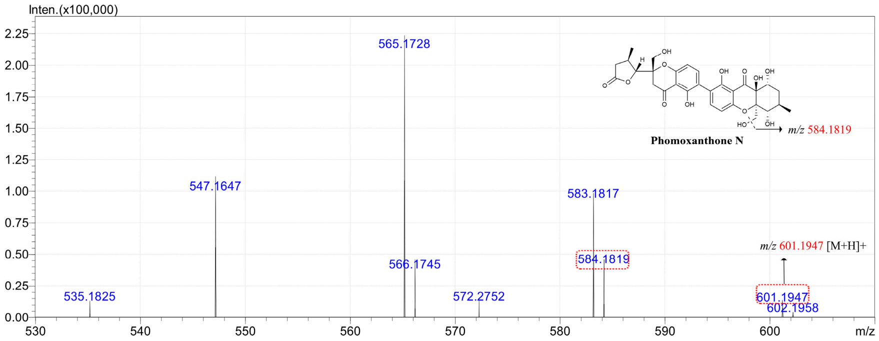

The (+)- MS/MS spectrum of compound **3** by the precursor of *m/z* 601.1947

B

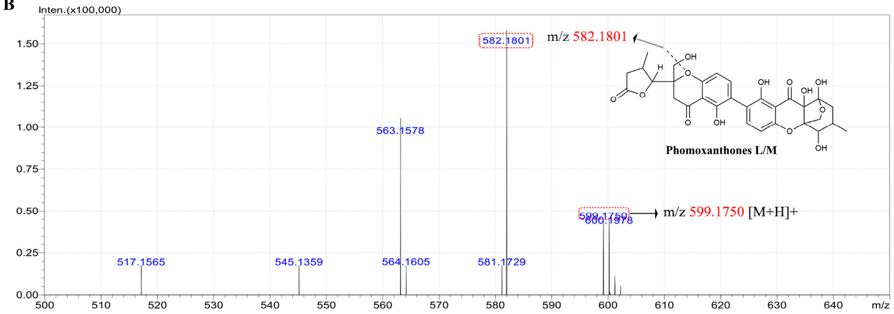

The (+)- MS/MS spectrum of compound **1/2** by the precursor of *m/z* 599.1750

C

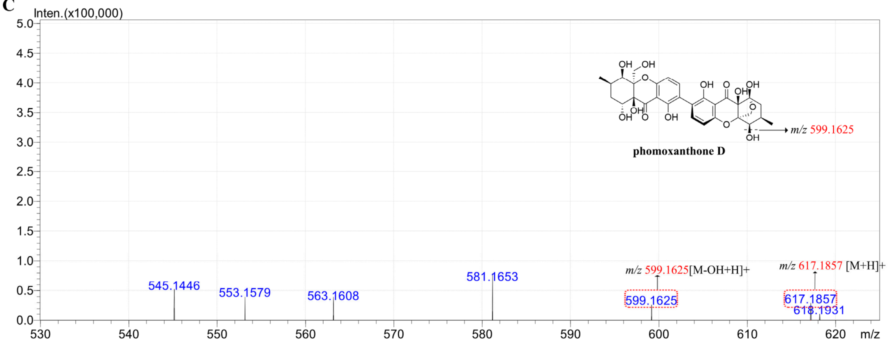

The (+)- MS/MS spectrum of compound **4** by the precursor of *m/z* 617.1857

Figure S4. The MS/MS spectrum and possible fragmentation patterns of compound **1**, **2**, **3** and **4**

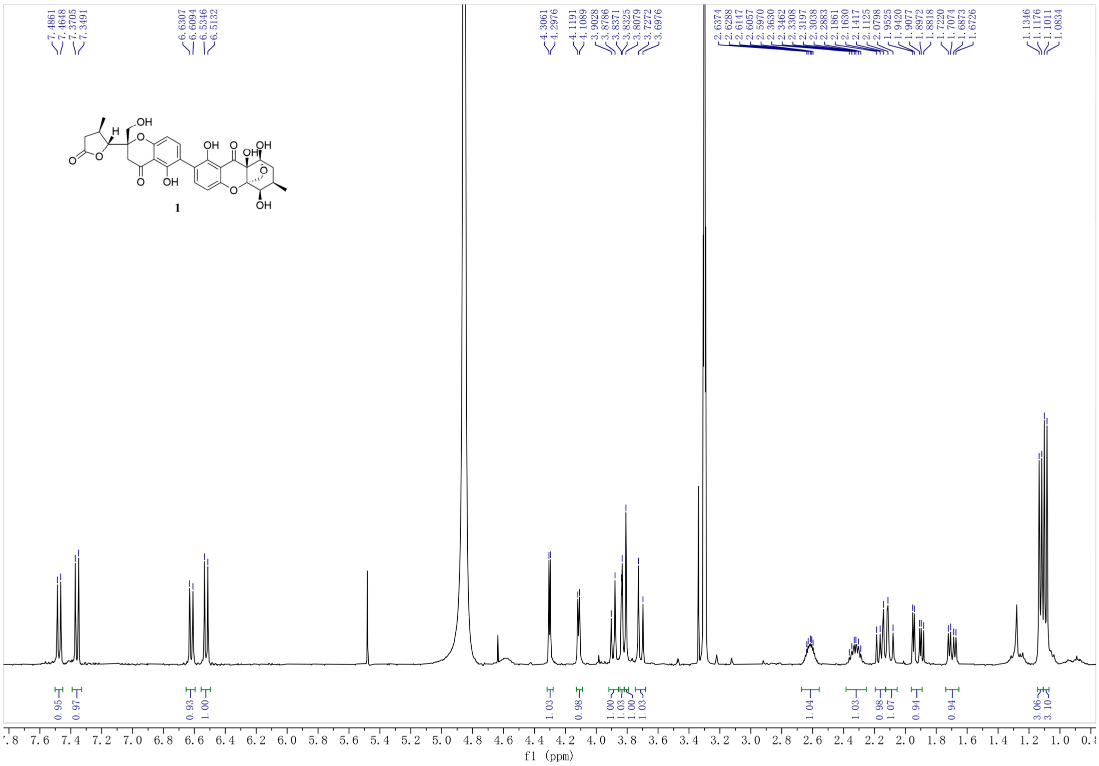

Figure S5. ^{1}H-NMR of (**1**)

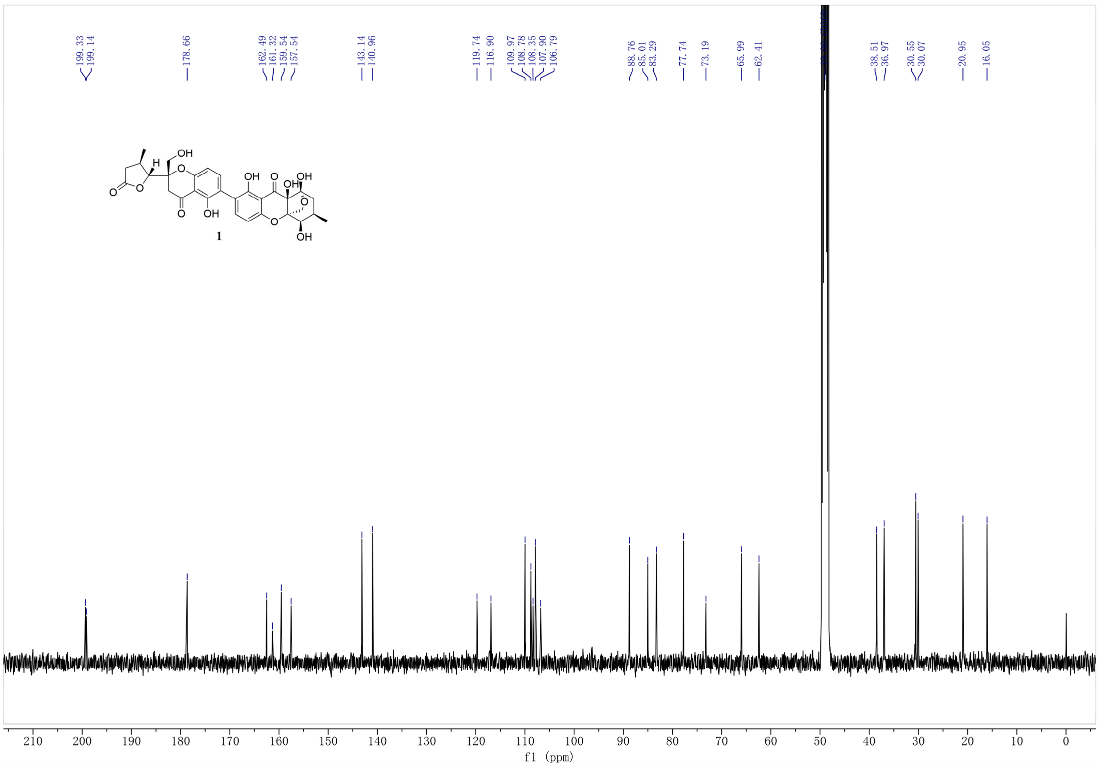

Figure S6. ^{13}C-NMR of (**1**)

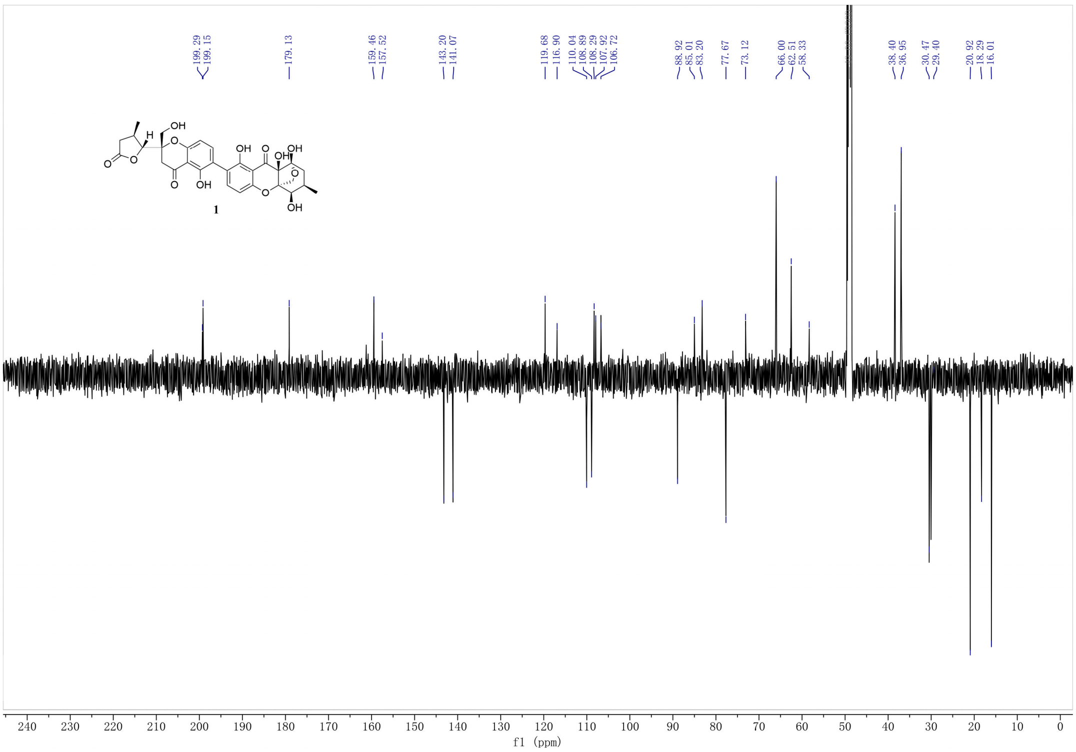

Figure S7. DEPT of (**1**)

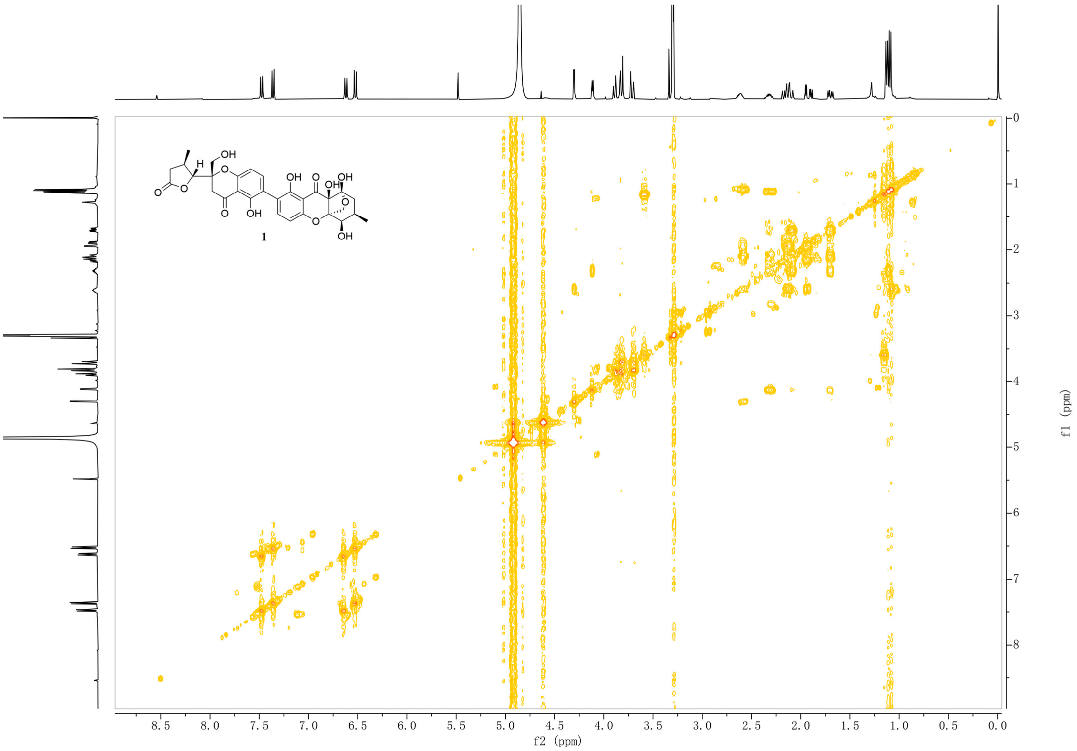

Figure S8. ^{1}H-^{1}H COSY of (**1**)

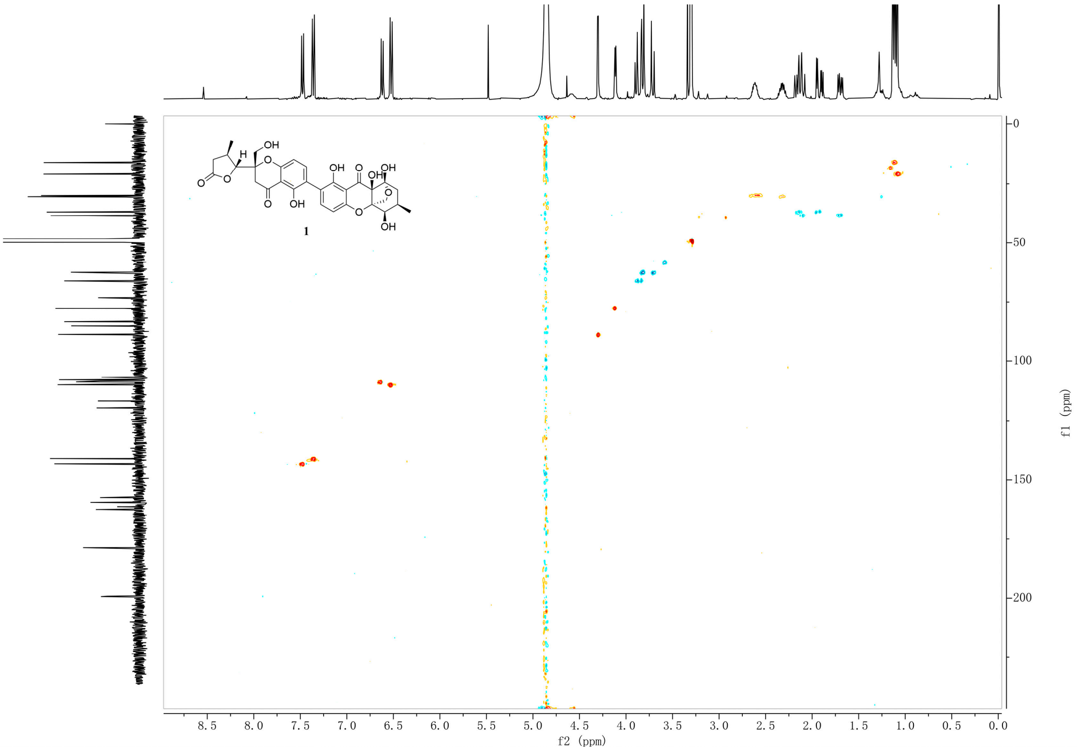

Figure S9. HSQC of (**1**)

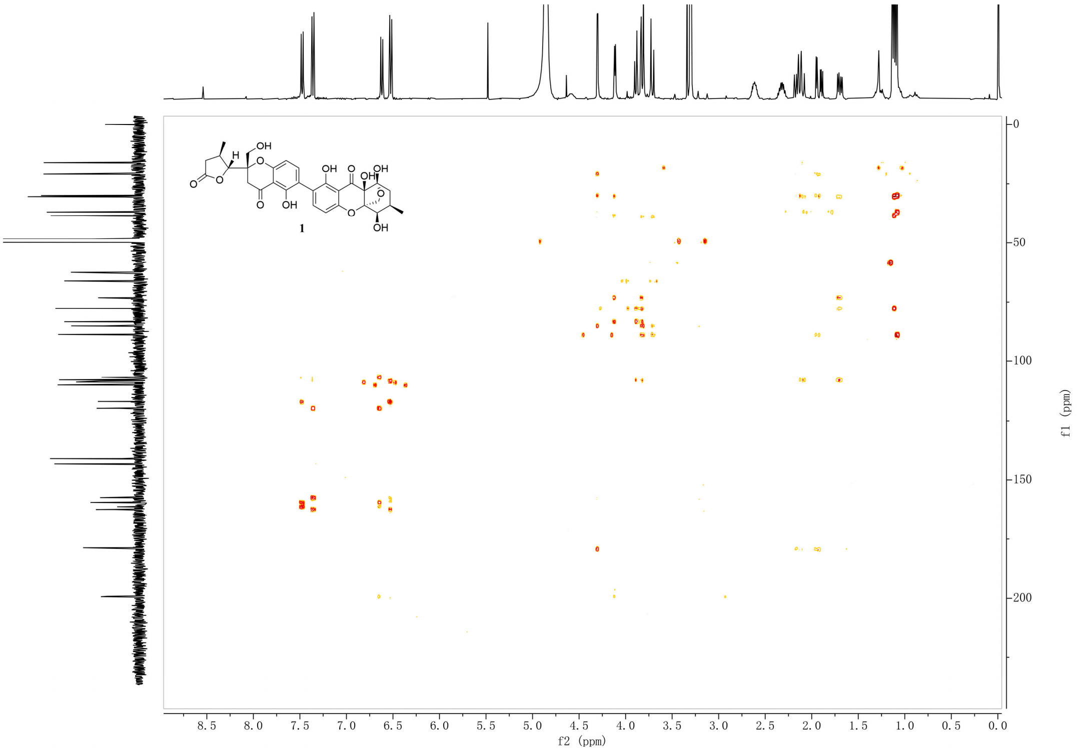

Figure S10. HMBC of (**1**)

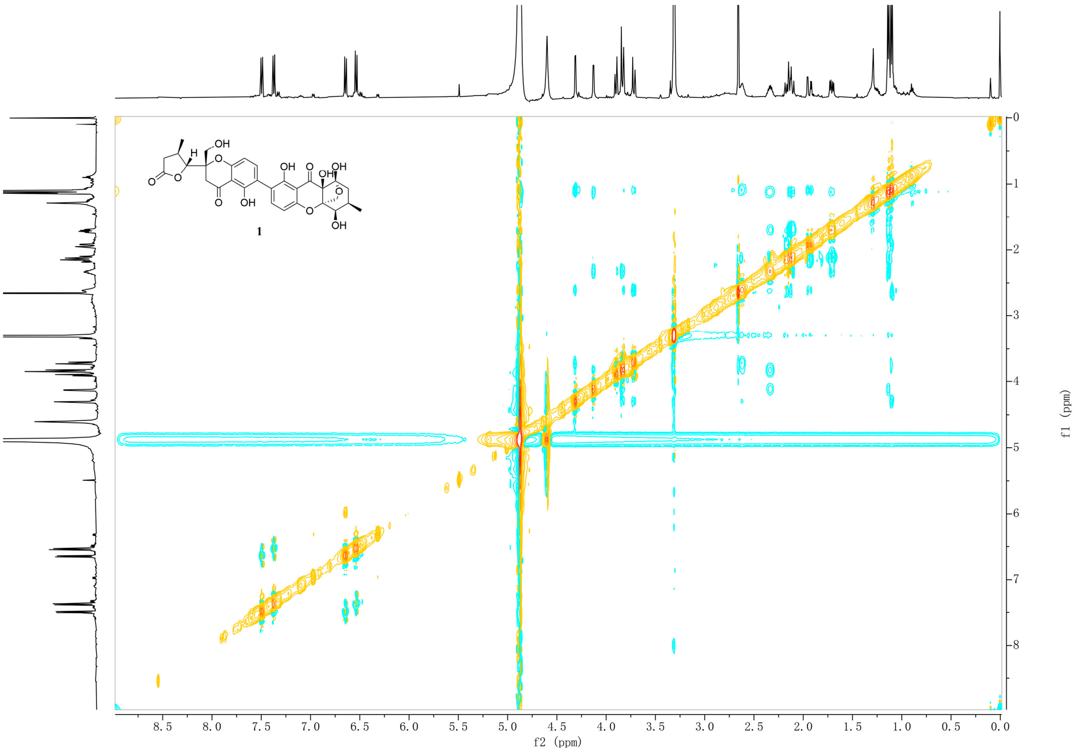

Figure S11. NOSEY of (**1**)

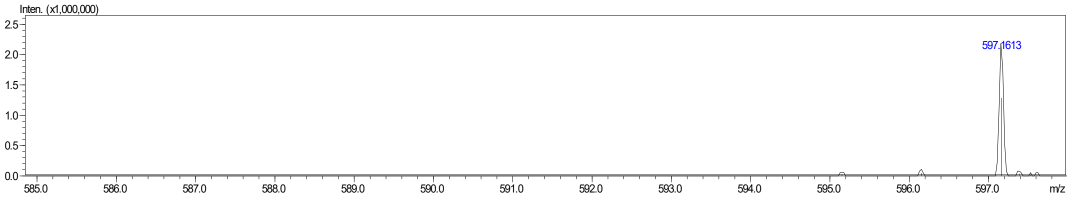

Figure S12. HR-ESI-MS of (**1**)

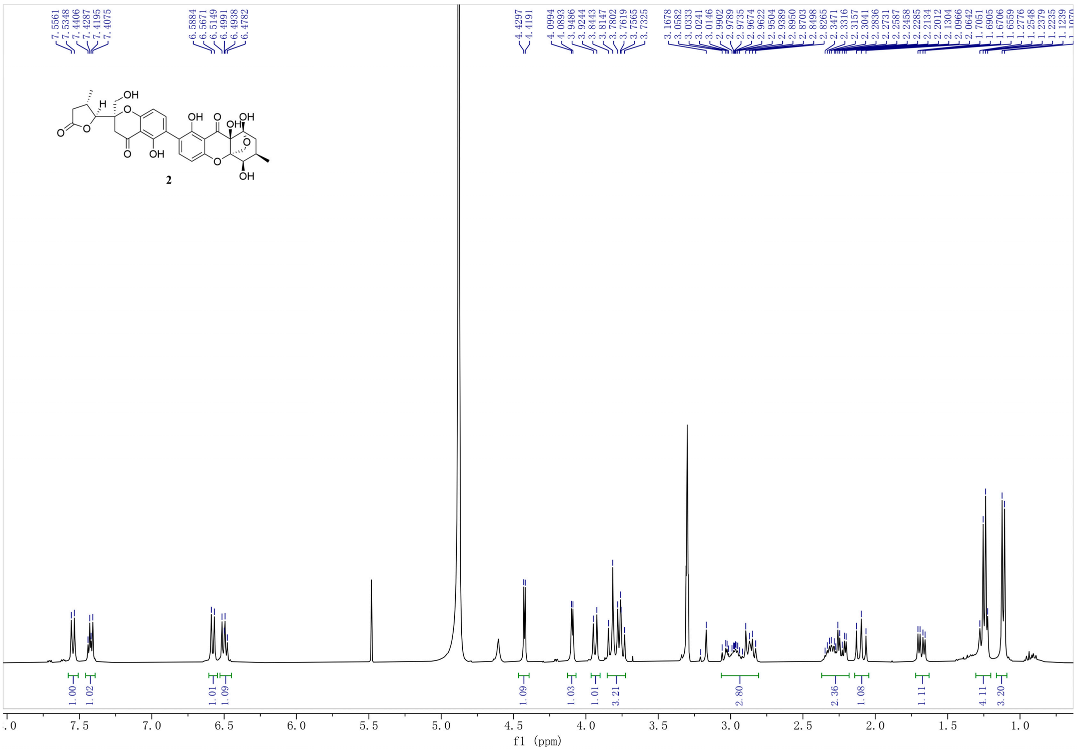

Figure S13. ^{1}H-NMR of (**2**)

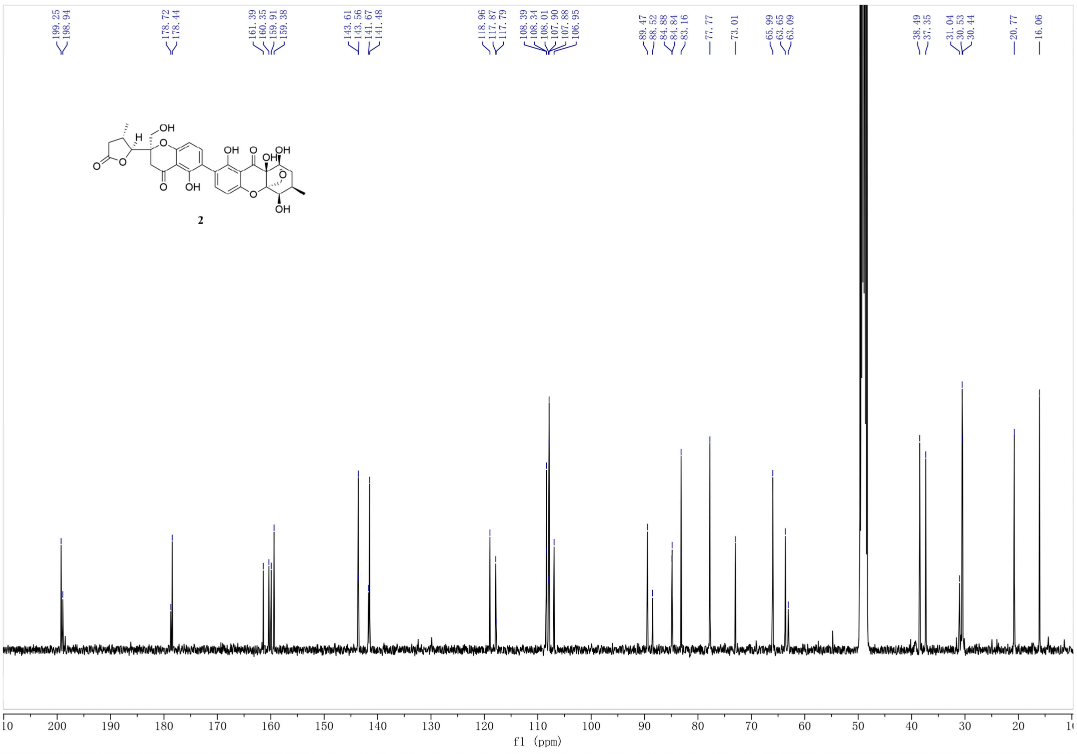

Figure S14. ^{13}C-NMR of (**2**)

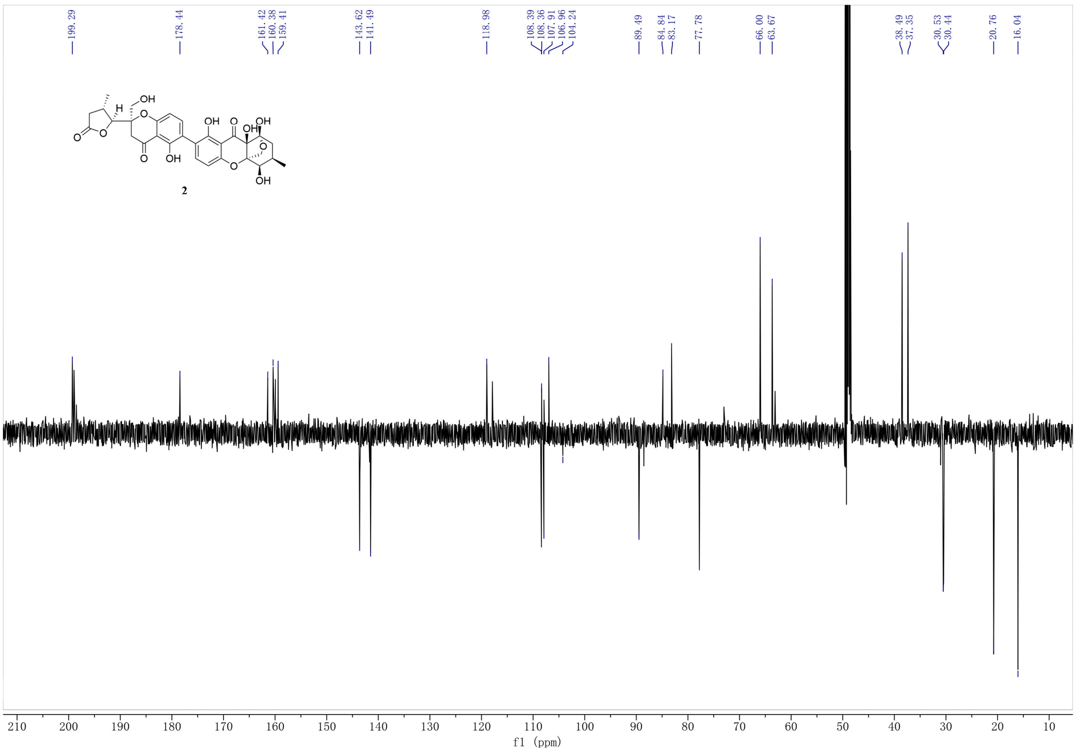

Figure S15. DEPT of (**2**)

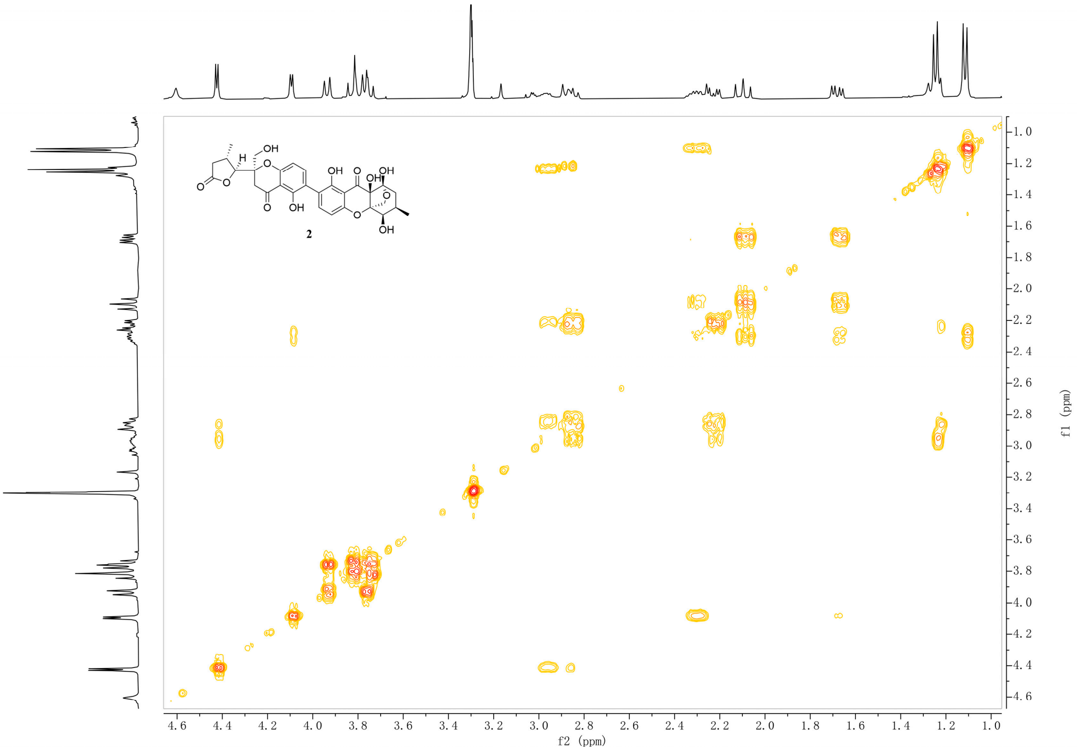

Figure S16. ^{1}H-^{1}H COSY of (**2**)

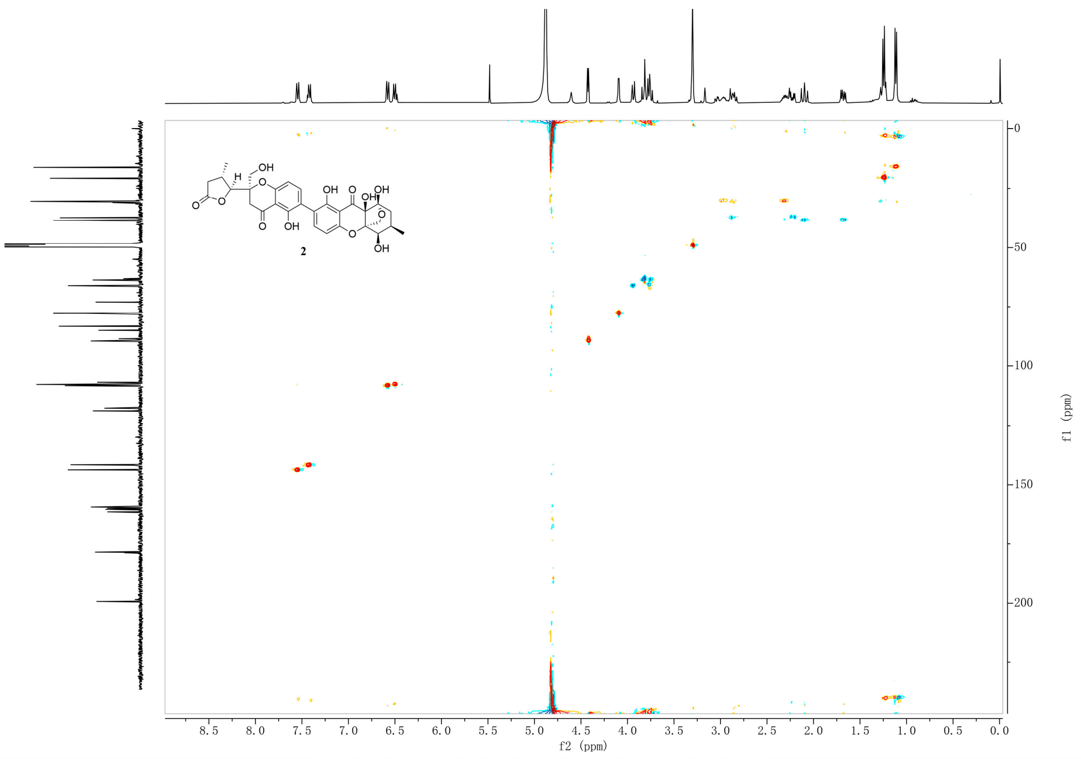

Figure S17. HSQC of (**2**)

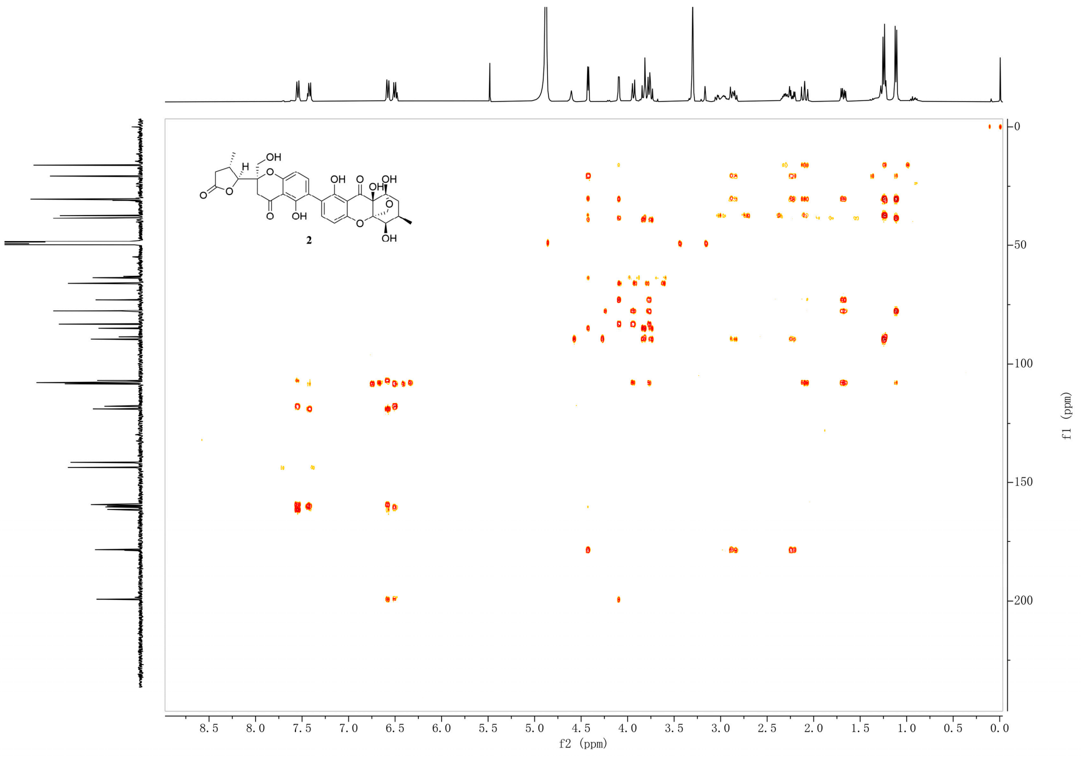

Figure S18. HMBC of (**2**)

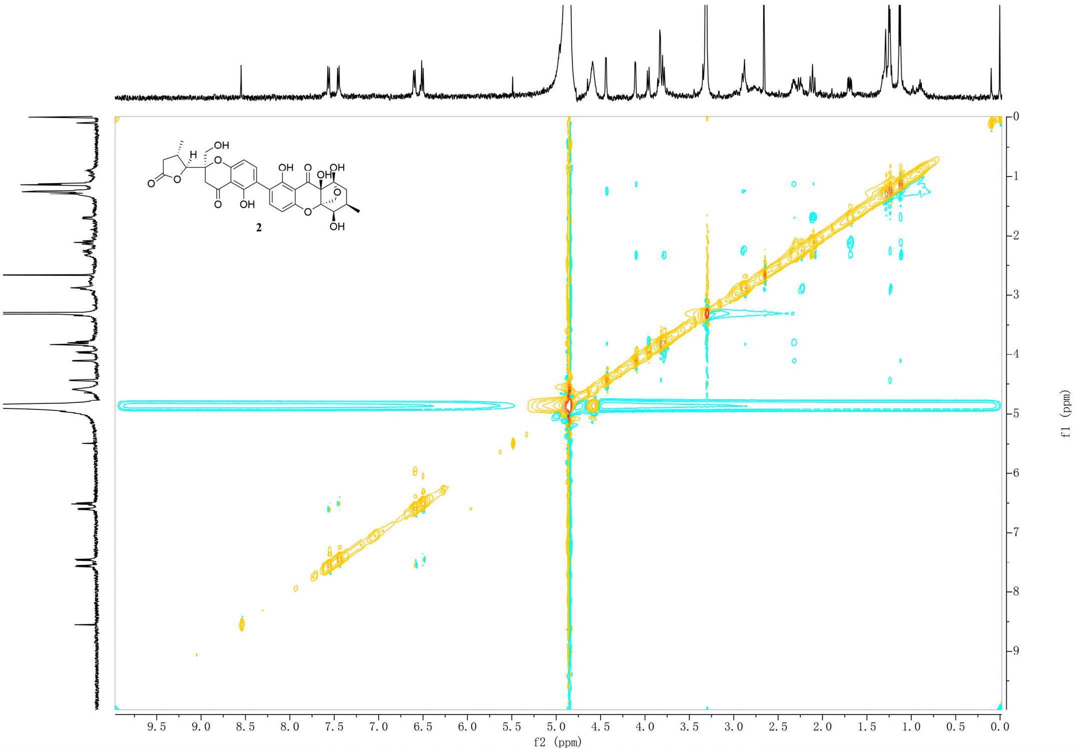

Figure S19. NOSEY of (**2**)

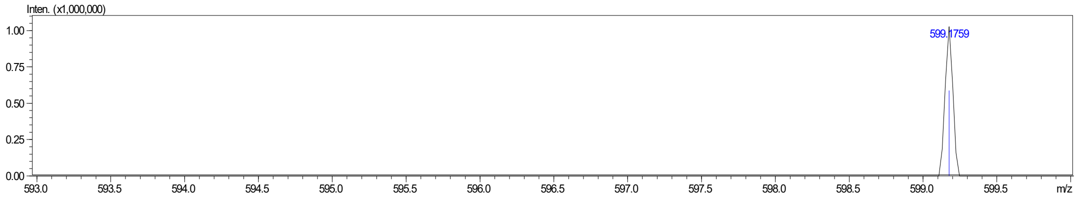

Figure S20. HR-ESI-MS of (**2**)

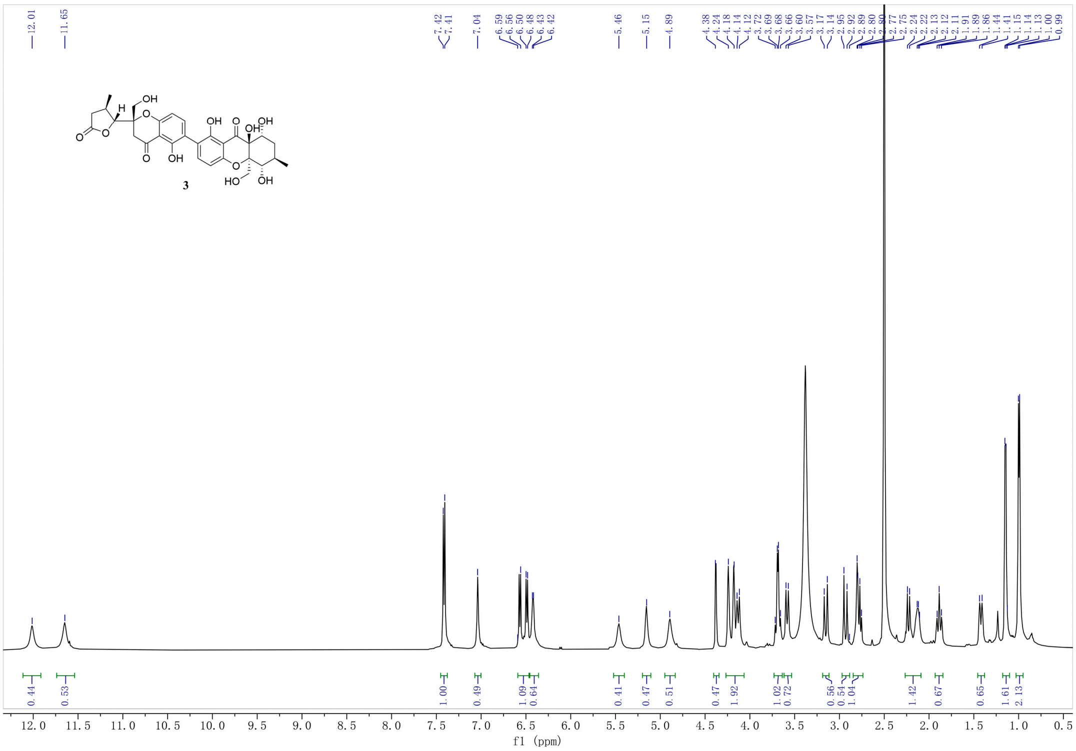

Figure S21. ^{1}H-NMR of (**3**)

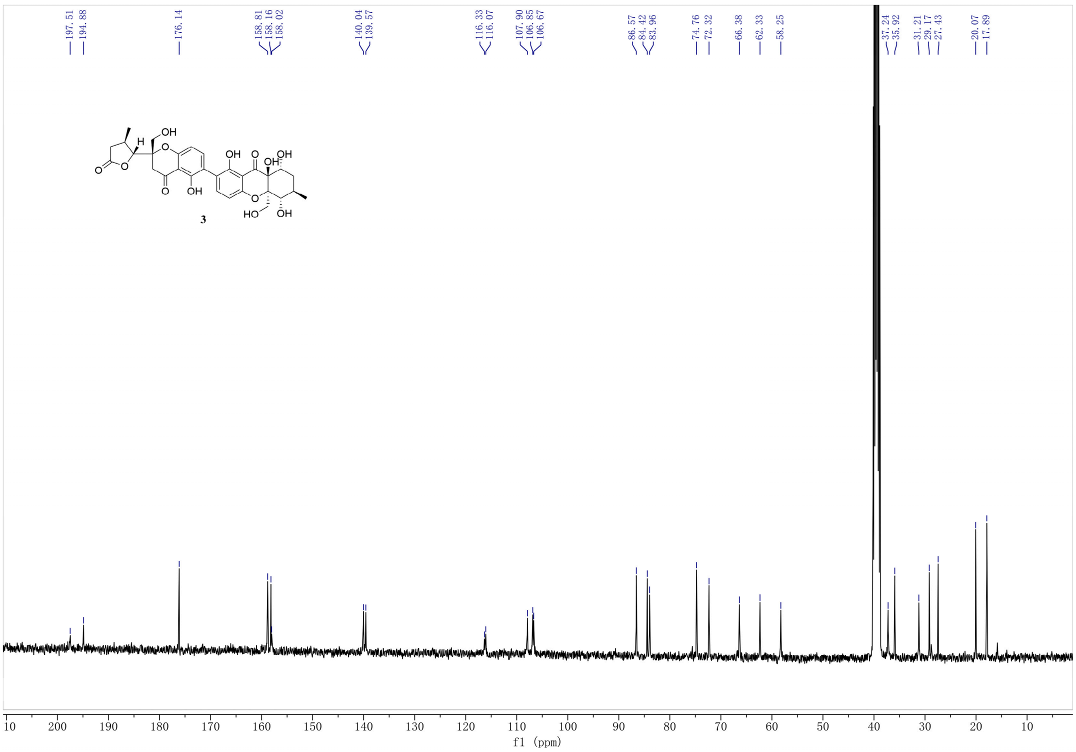

Figure S22. ^{13}C-NMR of (**3**)

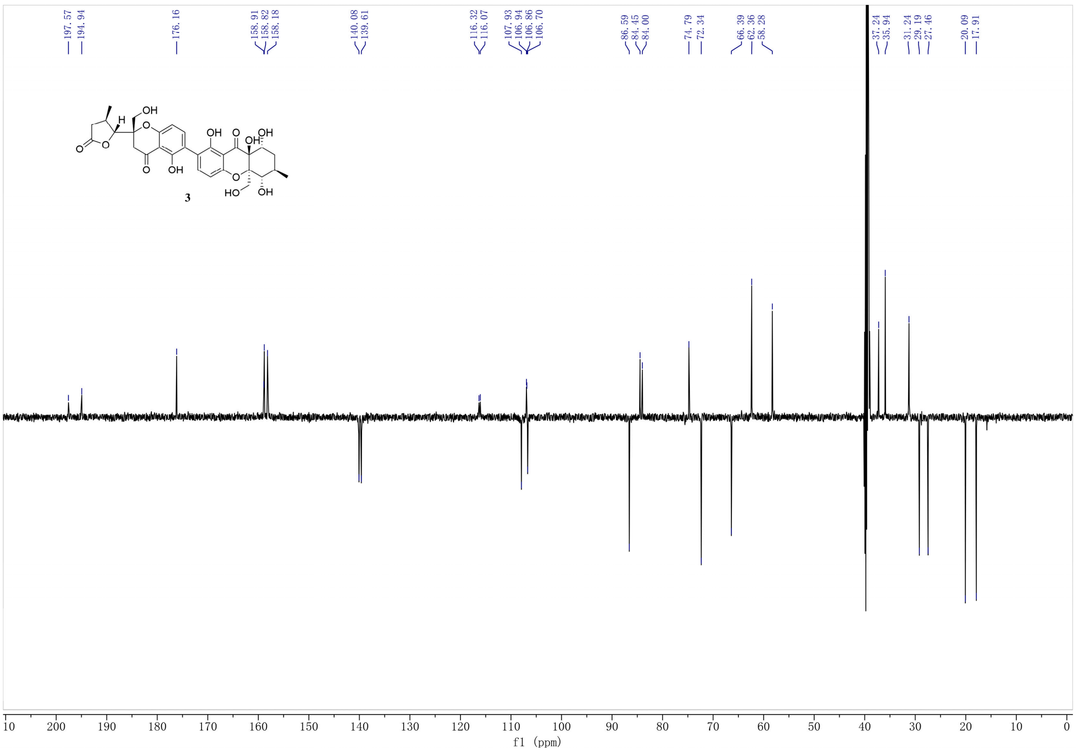

Figure S23. DEPT of (**3**)

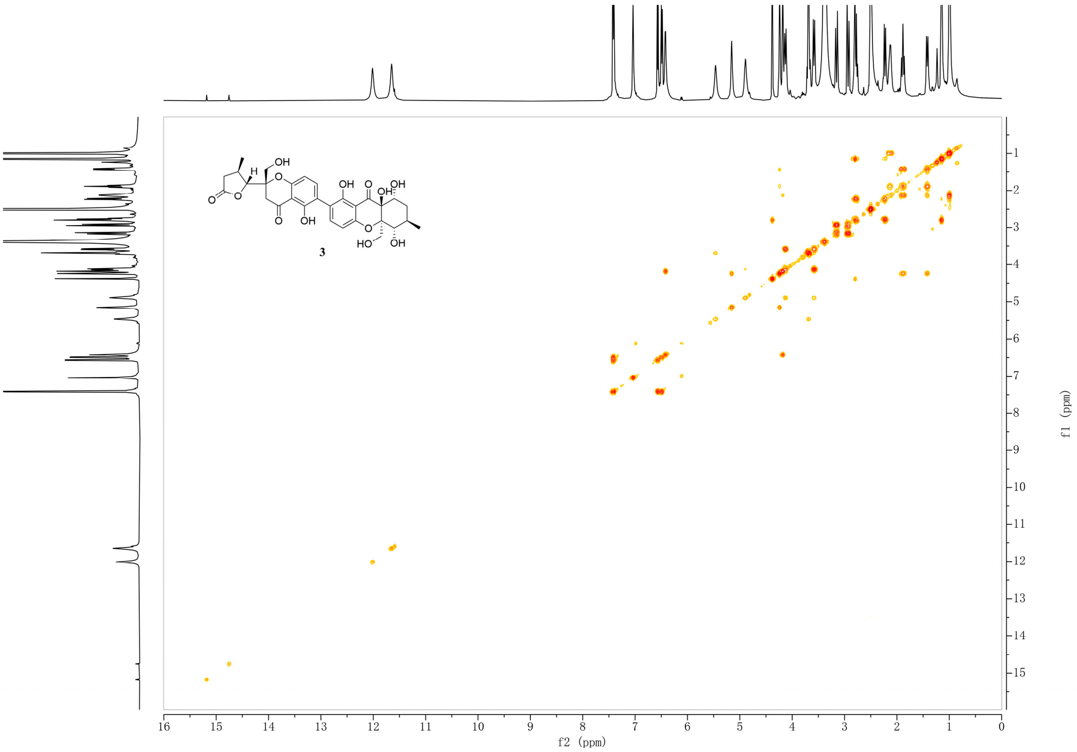

Figure S24. ^{1}H-^{1}H COSY of (**3**)

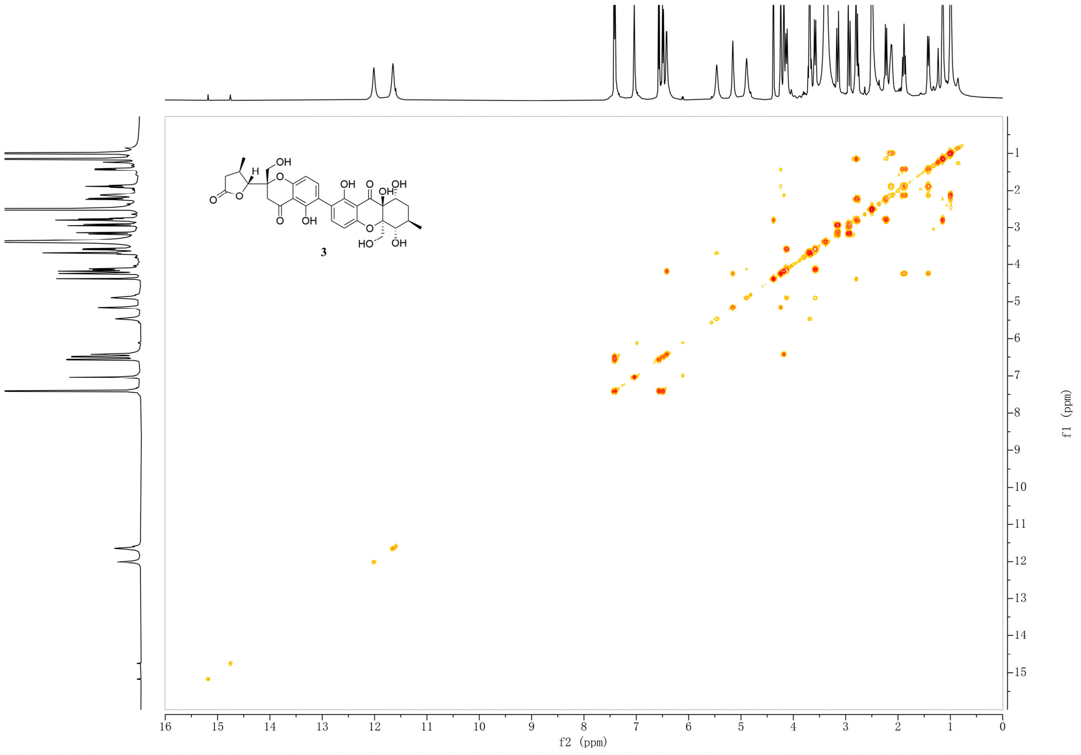

Figure S25. HSQC of (**3**)

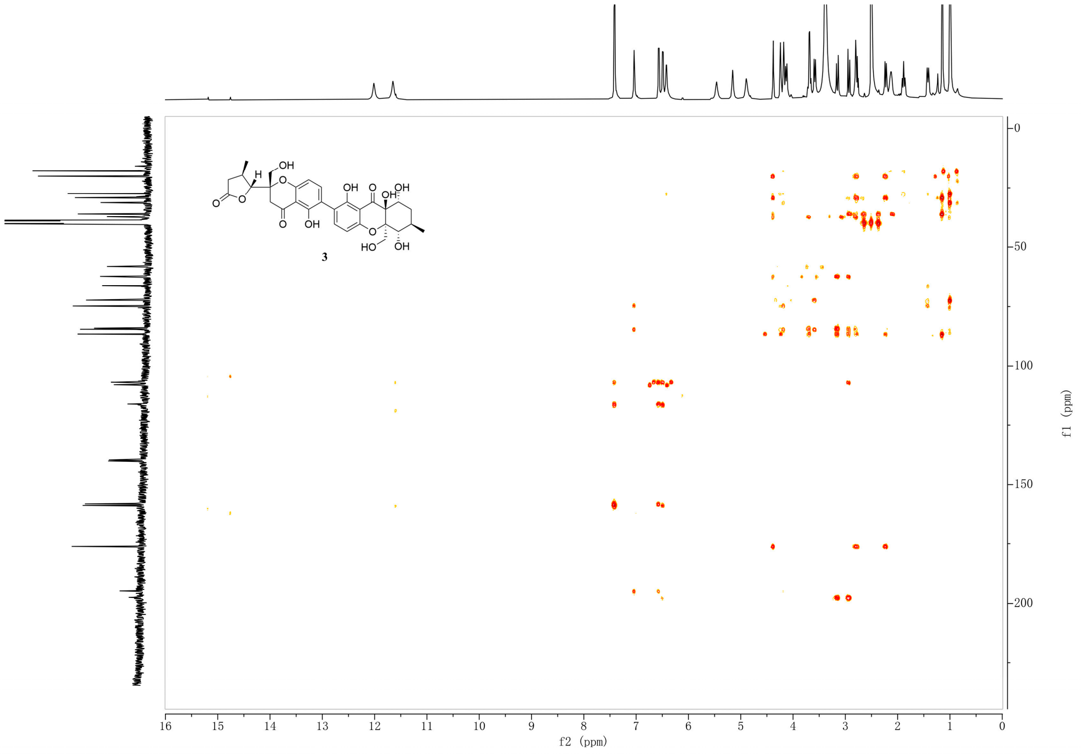

Figure S26. HMBC of (**3**)

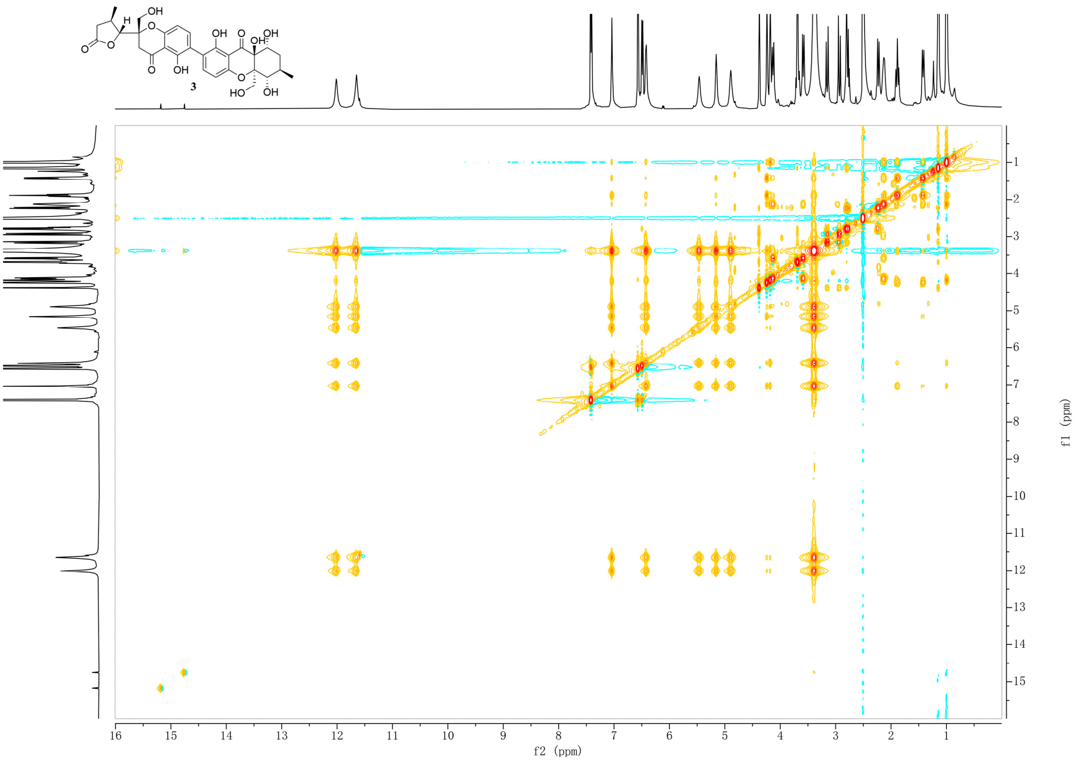

Figure S27. NOESY of (**3**)

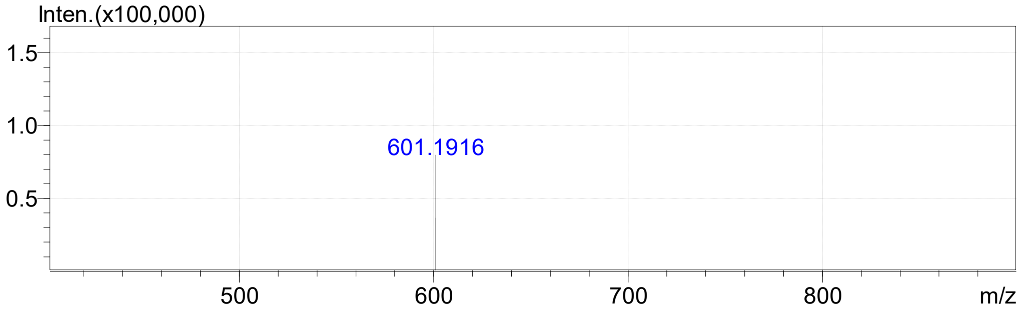

Figure S28. HR-ESI-MS of (**3**)

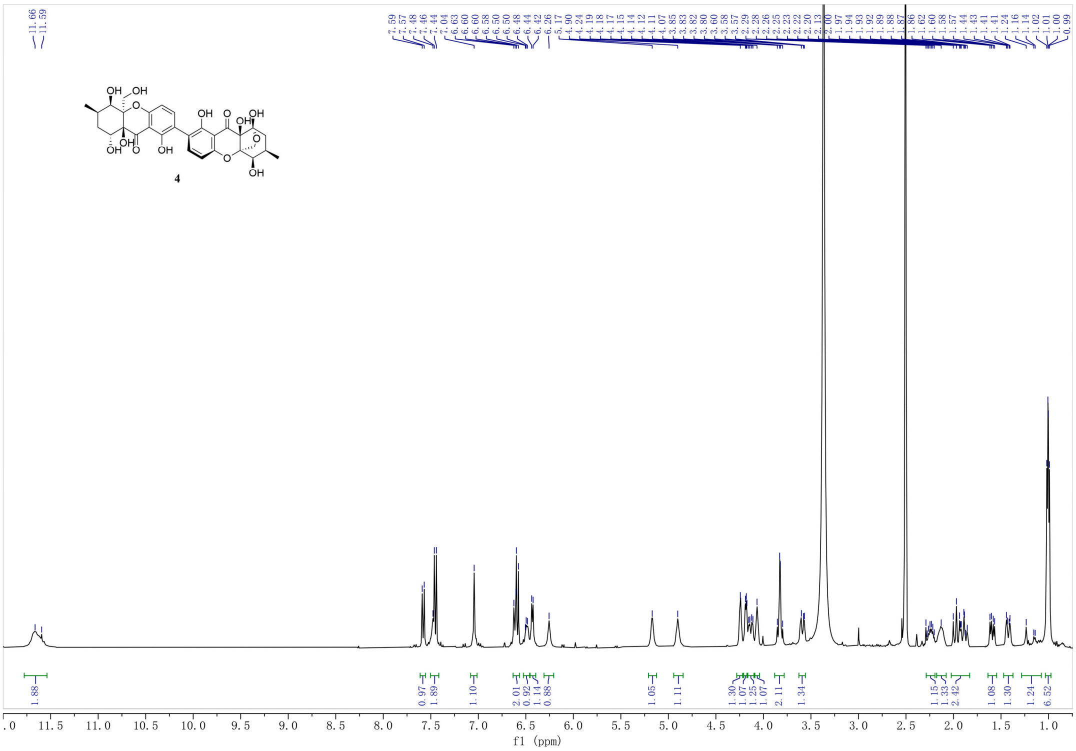

Figure S29. ^{1}H-NMR of (**4**)

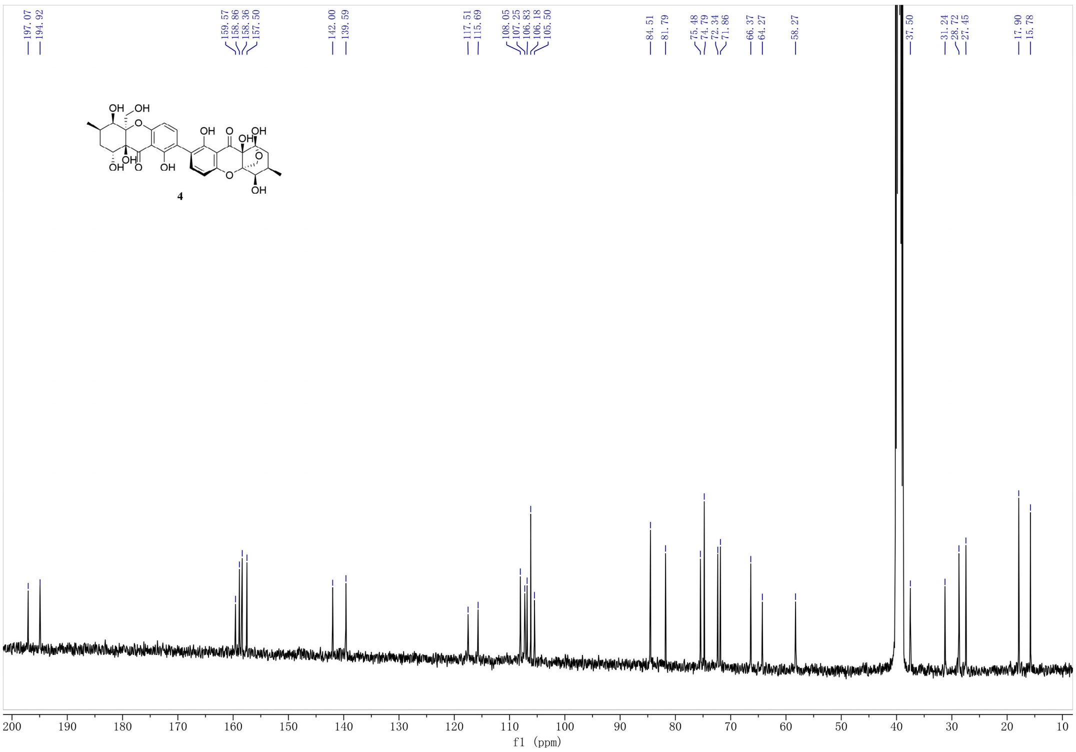

Figure S30. ^{13}C-NMR of (**4**)

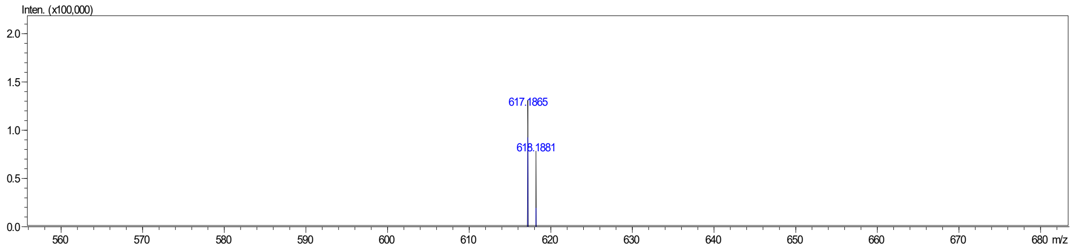

Figure S31. HR-ESI-MS of (**4**)

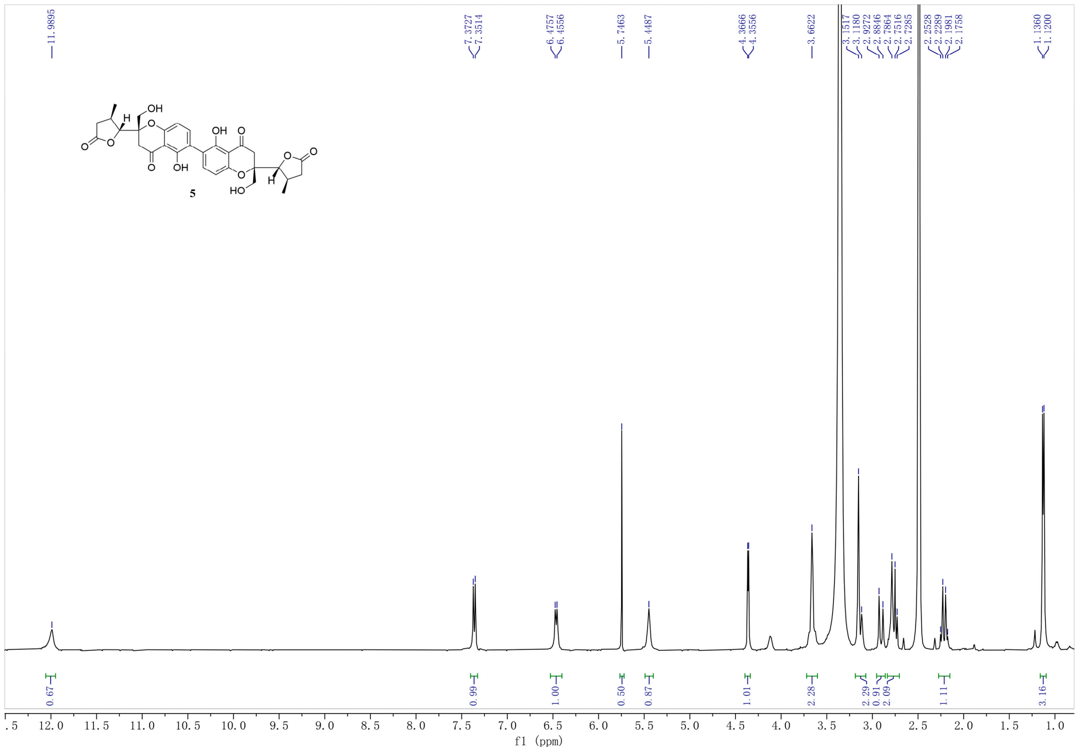

Figure S32. ¹H-NMR of (**5**)

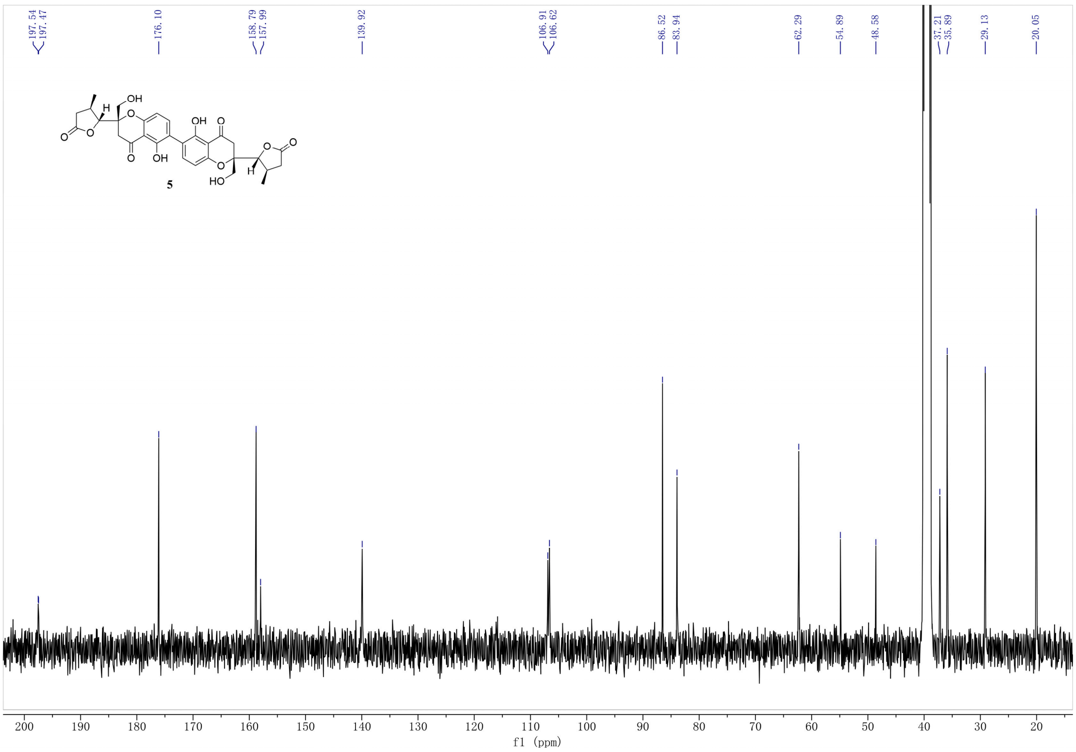

Figure S33. ^{13}C-NMR of (**5**)

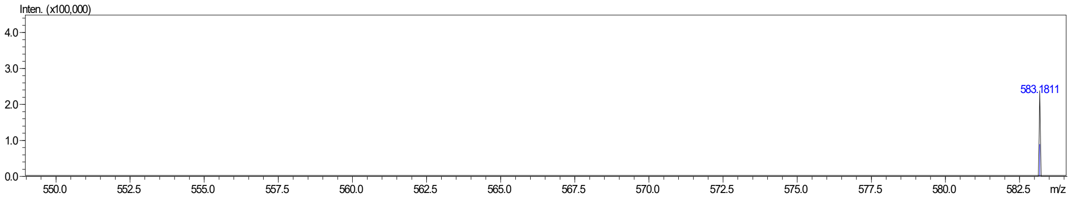

Figure S34. HR-ESI-MS of (**5**)

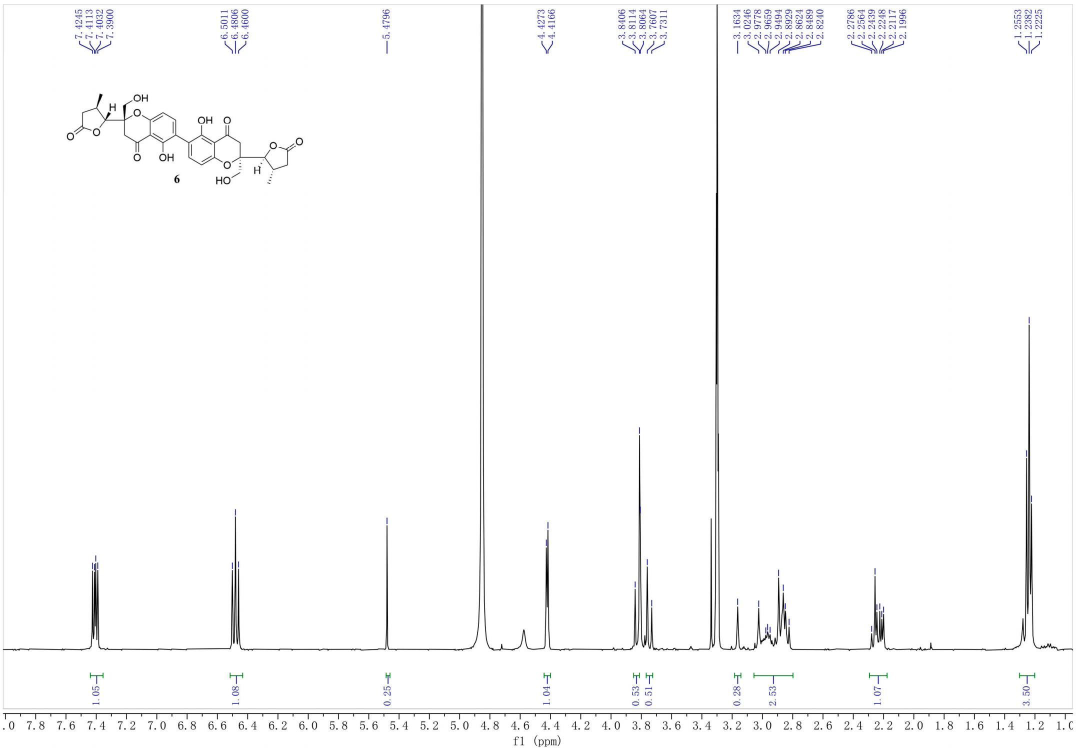

Figure S35. ^{1}H-NMR of (**6**)

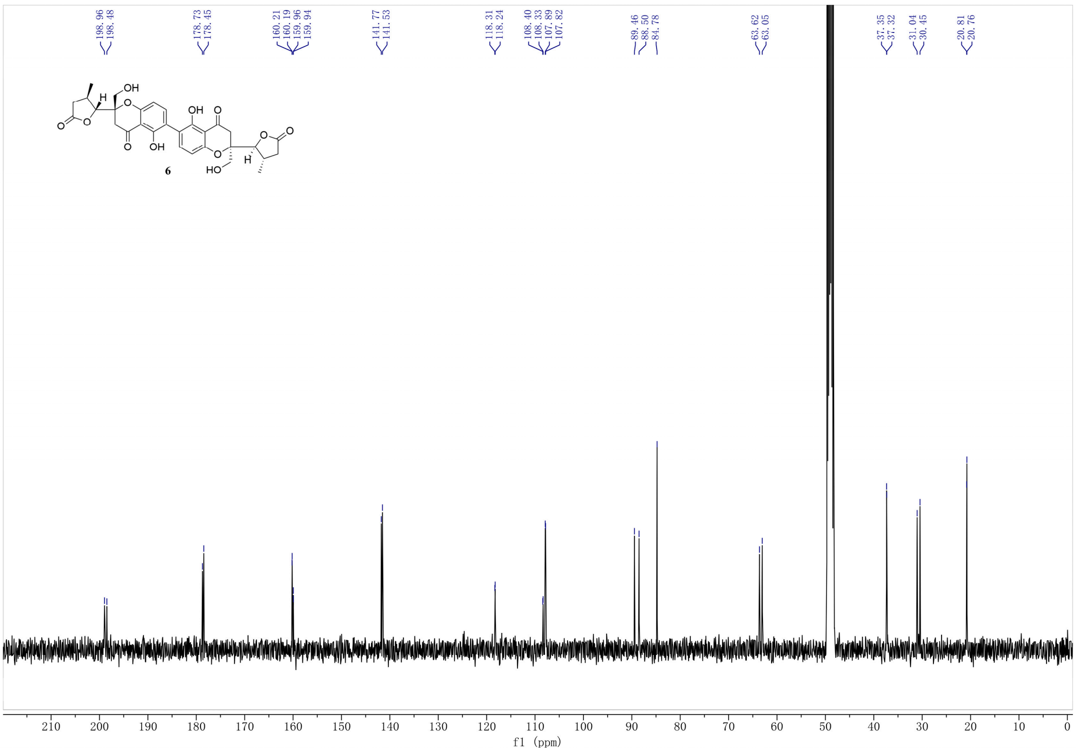

Figure S36. ^{13}C-NMR of (**6**)

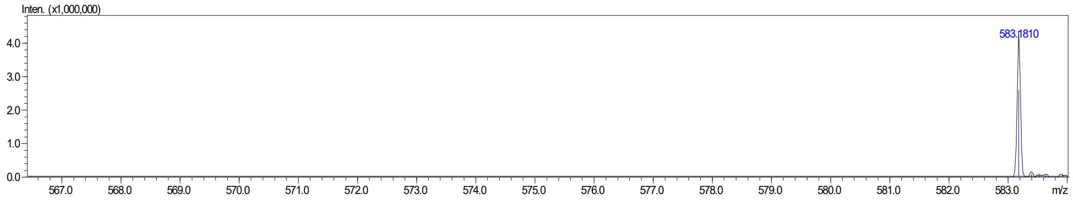

Figure S37. HR-ESI-MS of (**6**)

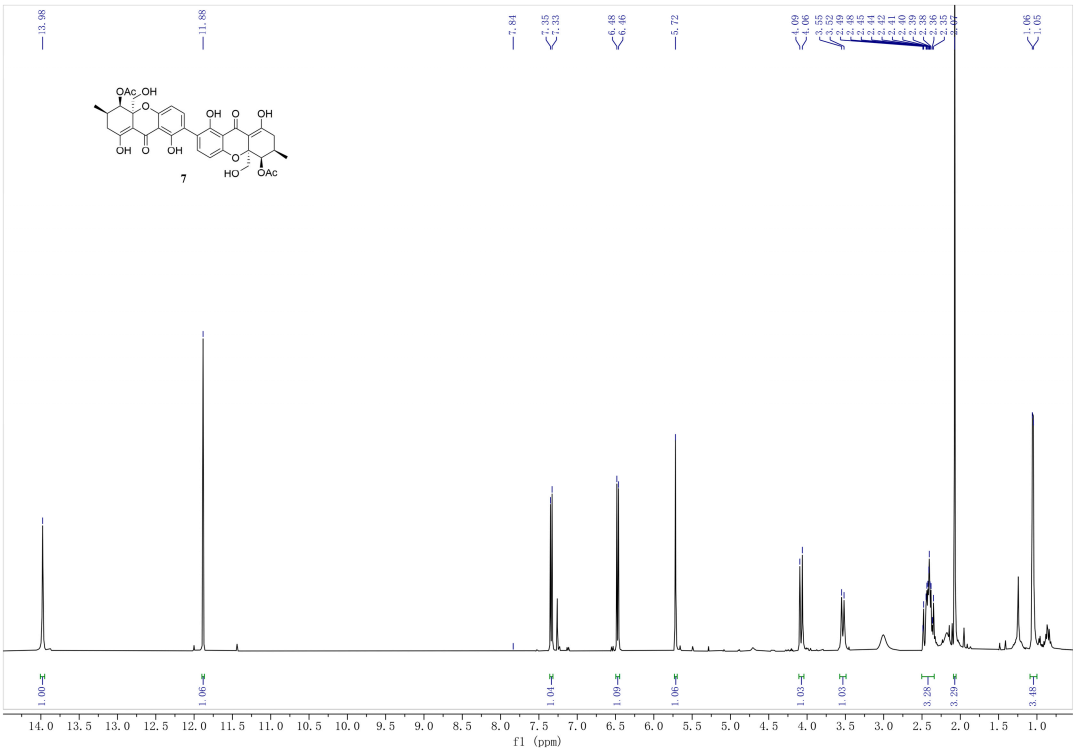

Figure S38. ^{1}H-NMR of (**7**)

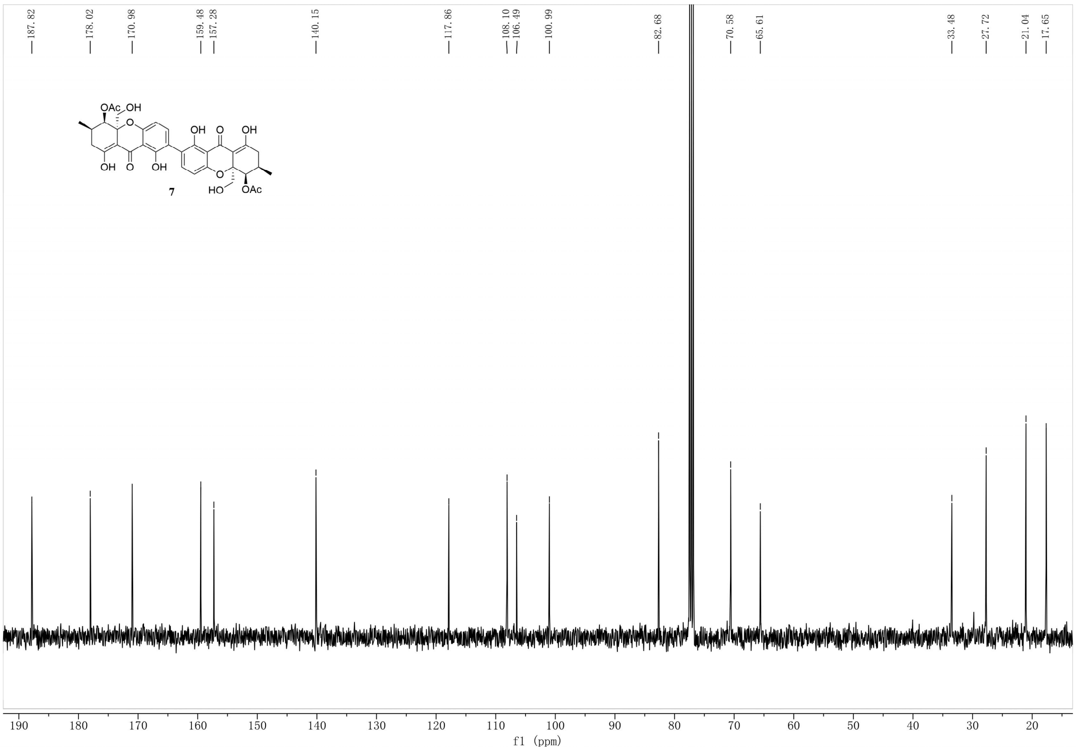

Figure S39. ^{13}C-NMR of (**7**)

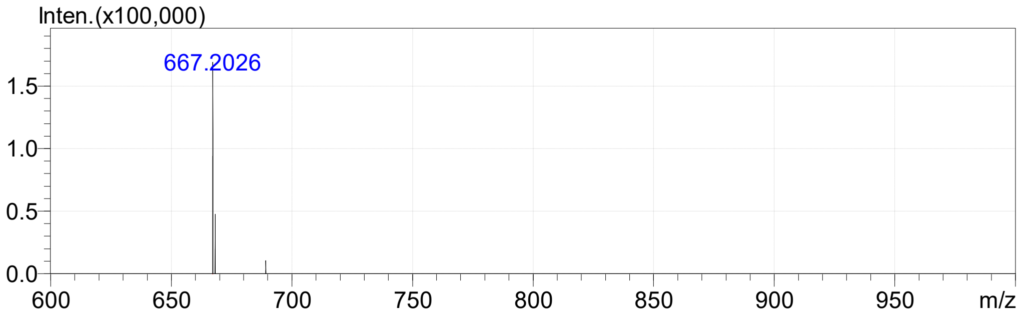

Figure S40. HR-ESI-MS of (**7**)

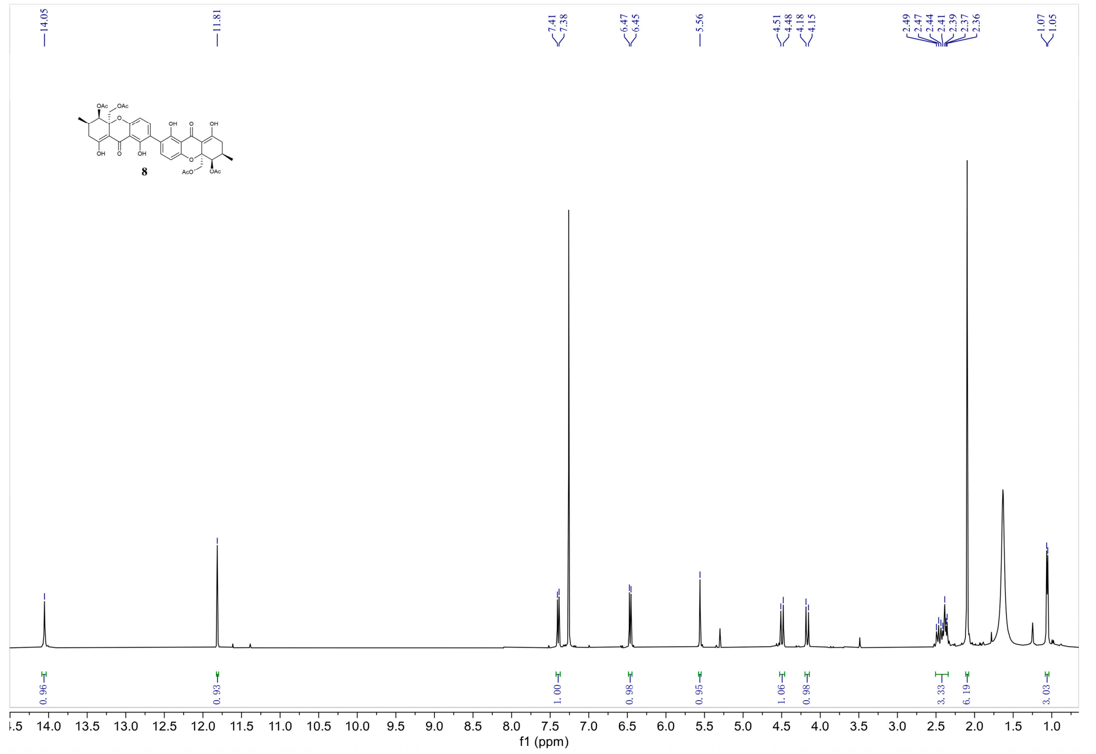

Figure S41. ^{1}H-NMR of (**8**)

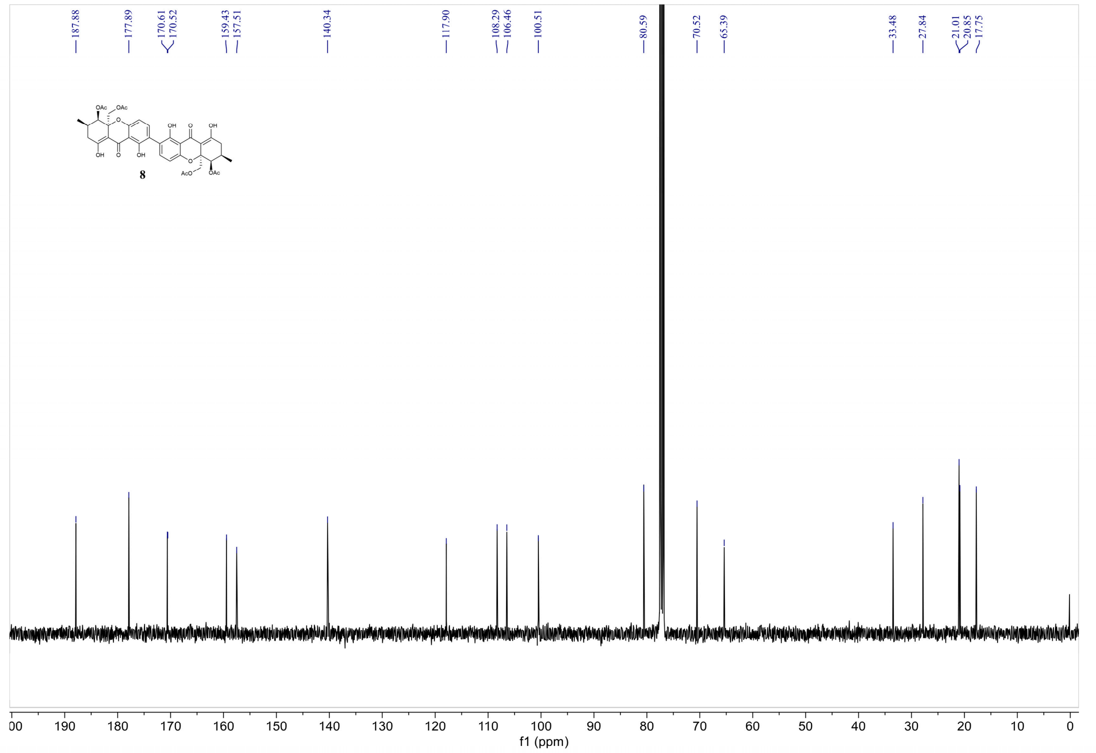

Figure S42. ^{13}C-NMR of (**8**)

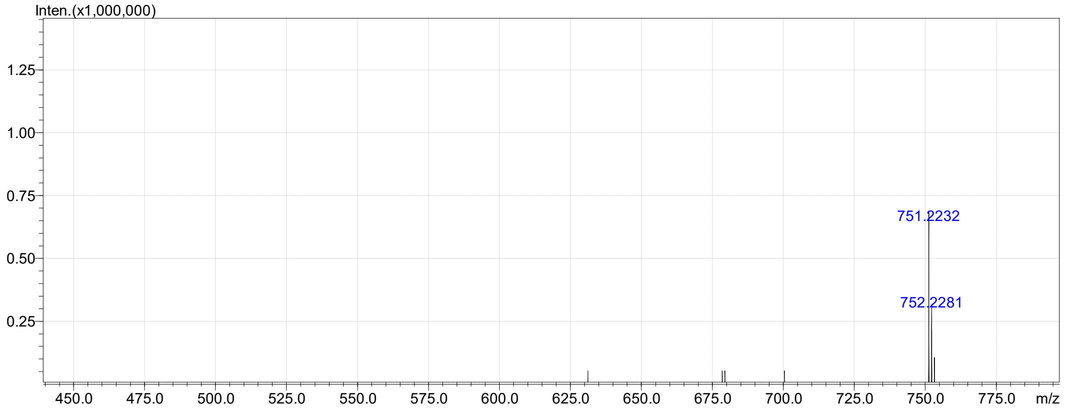

Figure S43. HR-ESI-MS of (**8**)

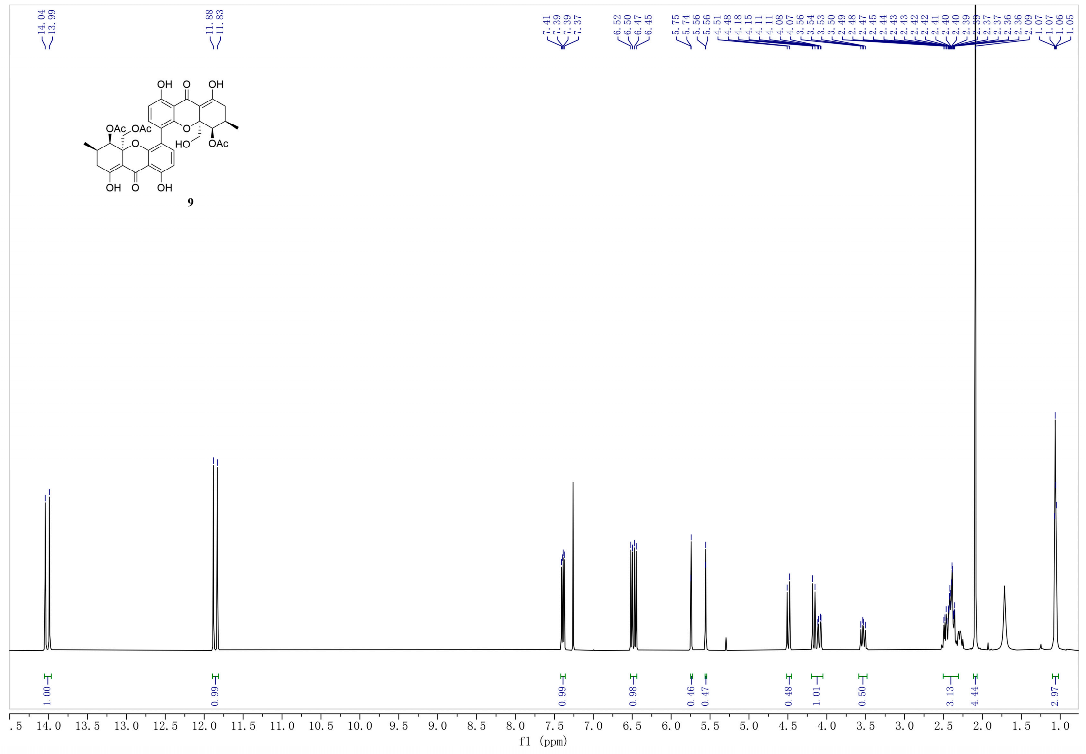

Figure S44. ^{1}H-NMR of (**9**)

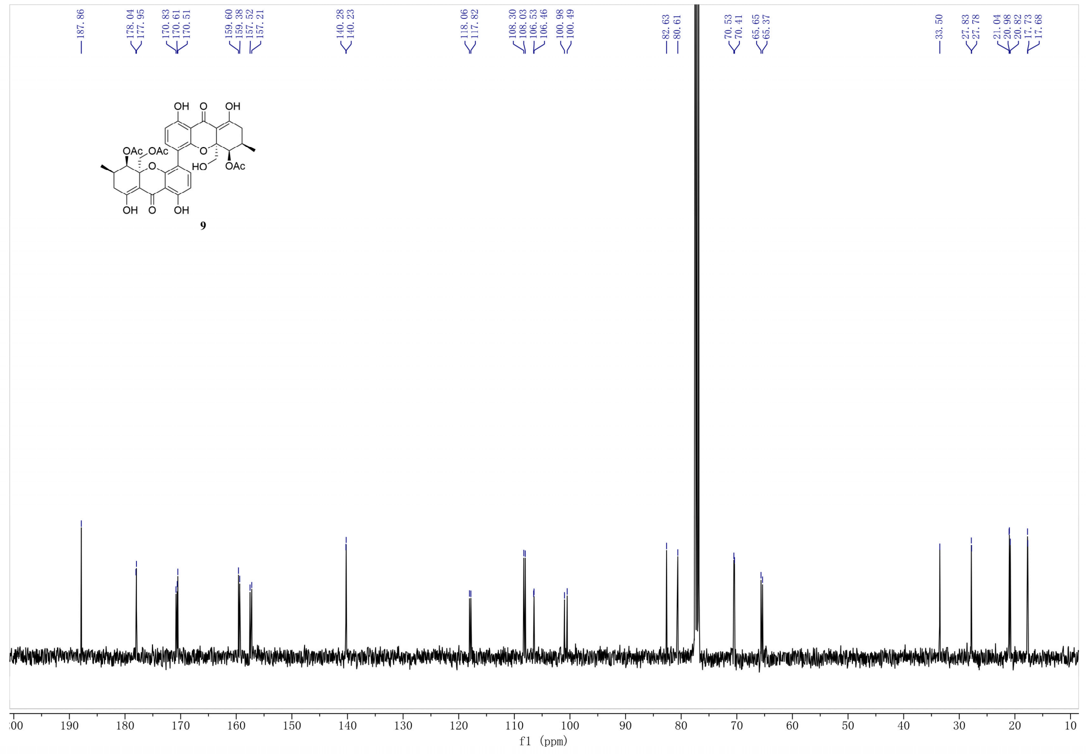

Figure S45. ^{13}C-NMR of (**9**)

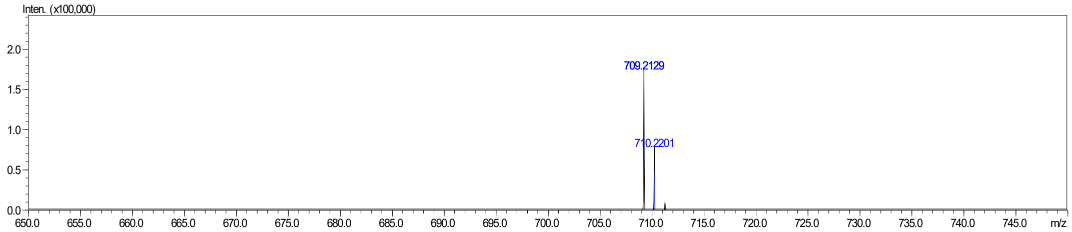

Figure S46. HR-ESI-MS of (**9**)

Figure S47. HPLC spectrum for the purity of tested compounds**.** HPLC chromatograms: C18 column (Agilent Technologies 10 mm×250 mm). Solvents: A, H_2O; B, MeOH. Linear gradient: 0 min,60% B; 40 min, 100% B. Temperature 25°C. Flow rate 2 mL/min. UV detection at λ = 210 nm.

Compound 1

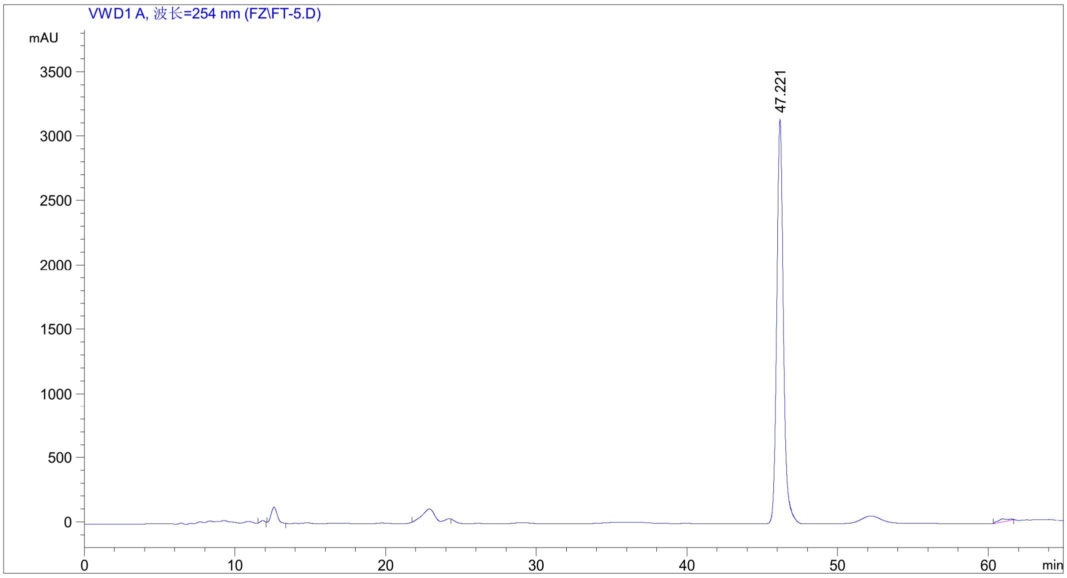

Peak #	Retention time [min]	Peak type	Peak width [min]	Peak area mAU *s	Peak height [mAU]	Peak area% %
1	47.221	BB	1.2593	1.20996e5	3106.93091	100.0000

Compound 2

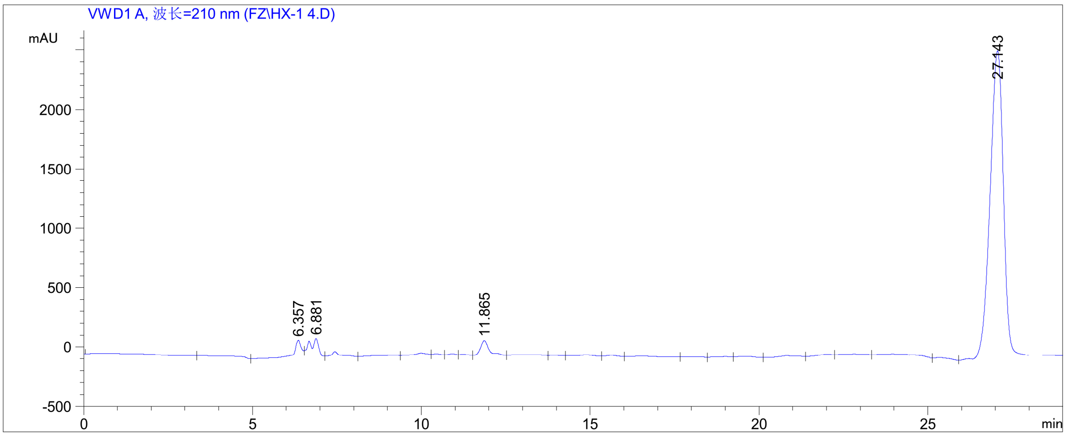

Peak #	Retention time [min]	Peak type	Peak width [min]	Peak area mAU *s	Peak height [mAU]	Peak area% %
1	6.357	VV	0.2441	2710.28540	155.47215	2.5016
2	6.881	VV	0.2796	3483.05005	170.15250	3.2149
3	11.865	VV	0.3394	3600.01855	149.40637	3.3228
4	27.143	VV	0.4409	7.39464e4	2612.70605	90.9607

Compound 3

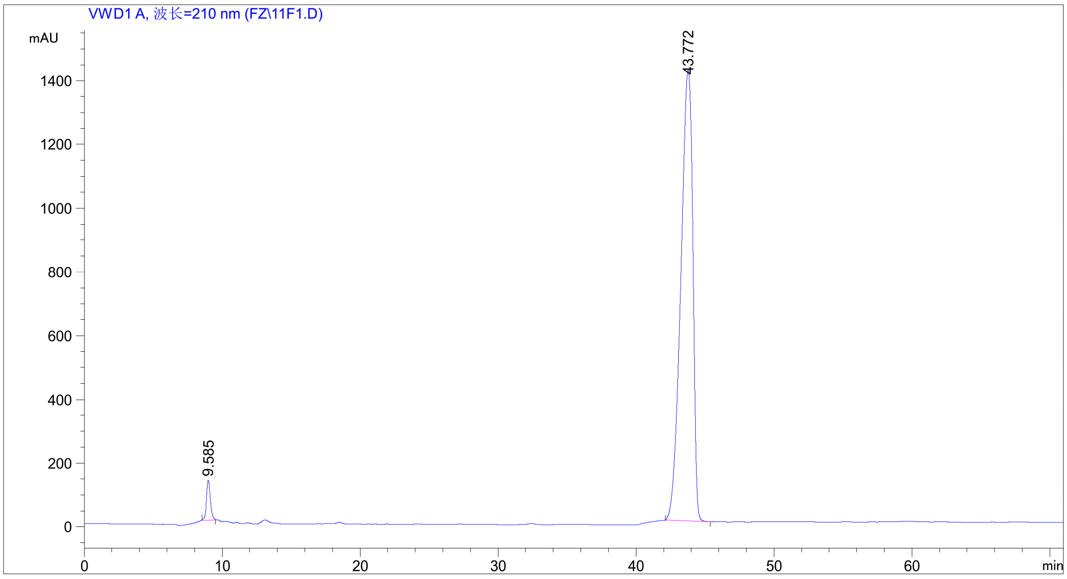

Peak	Retention time	Peak type	Peak width	Peak area	Peak height	Peak area%
1	9.585	BB	0.1490	1217.36353	126.09512	3.5773
2	43.772	BB	0.9359	3.28125e4	1473.50134	96.4227

Compound 4

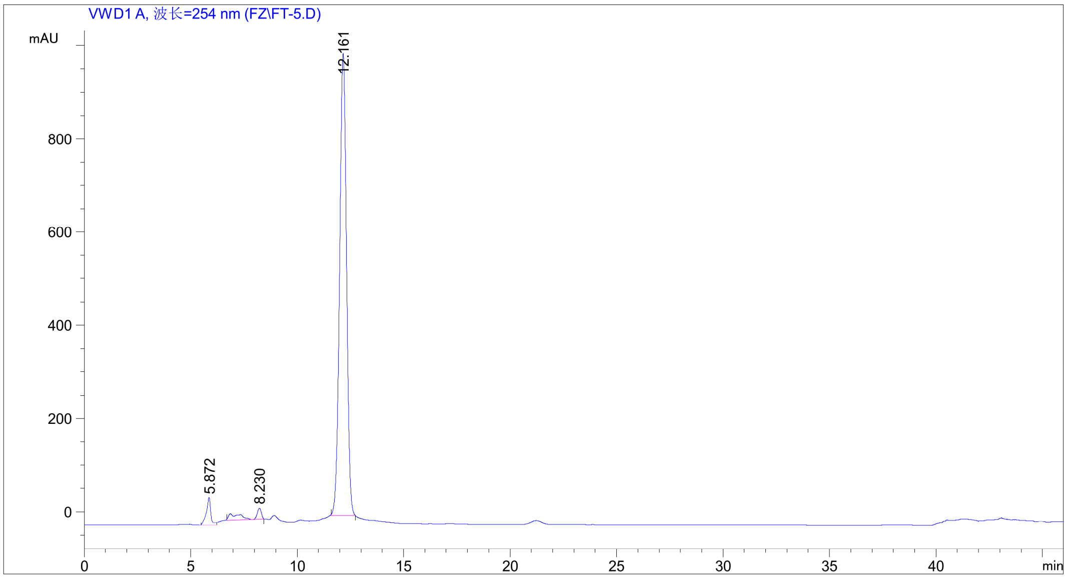

Peak #	Retention time [min]	Peak type	Peak width [min]	Peak area mAU *s	Peak height [mAU]	Peak area% %
1	5.872	BH	0.2014	1132.56433	53.58827	4.6731
2	8.230	HB S	0.4741	821.06448	23.47560	3.3519
3	12.161	BB S	0.3498	2.22909e4	990.78613	91.9750

Compound 5

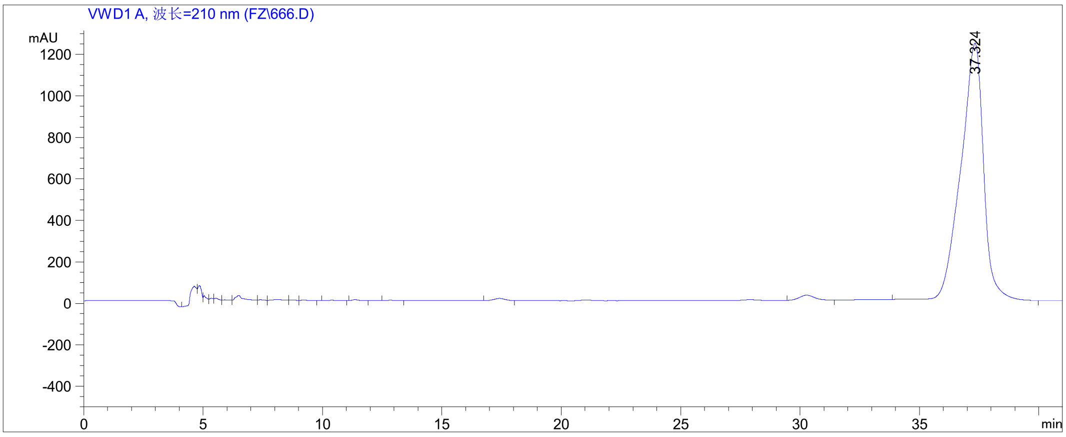

Peak	Retention time	Peak type	Peak width	Peak area	Peak height	Peak area%
1	37.324	BB	0.9588	8.20048e4	1238.60803	100.0000

Compound 6

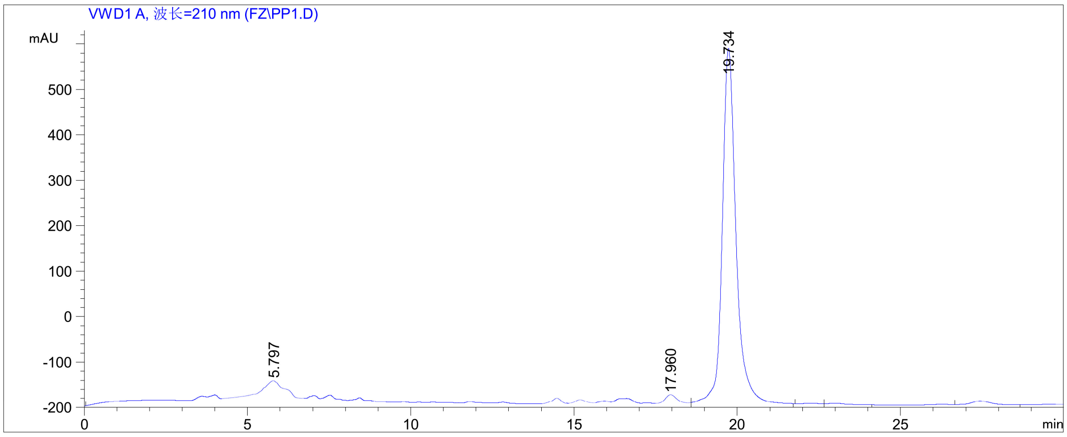

Peak #	Retention time [min]	Peak type	Peak width [min]	Peak area mAU *s	Peak height [mAU]	Peak area% %
1	5.797	VV	0.8172	2295.15214	59.36319	8.5691
2	17.960	VV	0.4163	932.30321	35.99451	3.4808
3	19.734	VB	0.4486	2.35566e4	786.22247	87.9501

Compound 7

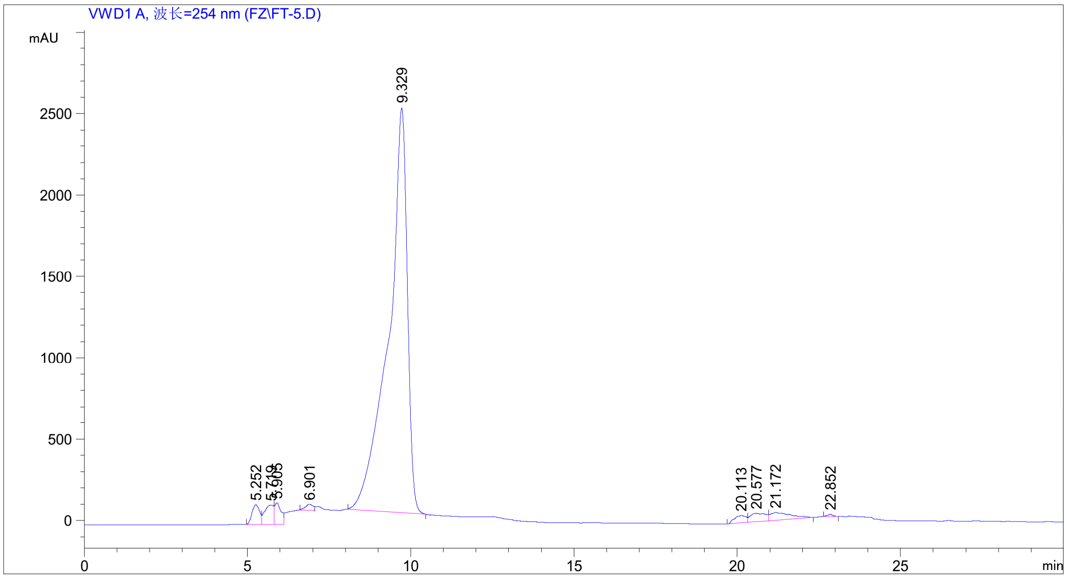

Peak #	Retention time [min]	Peak type		Peak width [min]	Peak area mAU *s	Peak height [mAU]	Peak area% %
1	5.252	BV		0.2374	1921.10400	120.19498	1.1893
2	5.719	VV		0.2798	2198.30981	118.07137	1.3609
3	5.905	VH		0.1942	1846.34888	131.64180	1.1430
4	6.901	HH	S	0.2324	563.54315	35.38733	0.3489
5	9.329	BB	S	0.5536	9.90195e4	2485.68408	92.9052
6	20.113	BV		0.2920	1095.53711	45.34671	0.6782
7	20.577	VV		0.4129	1690.26416	50.99595	1.0464
8	21.172	VB		0.4979	1972.24915	47.89462	1.2210
9	22.852	BB		0.1510	172.99254	15.27656	0.1071

Compound 8

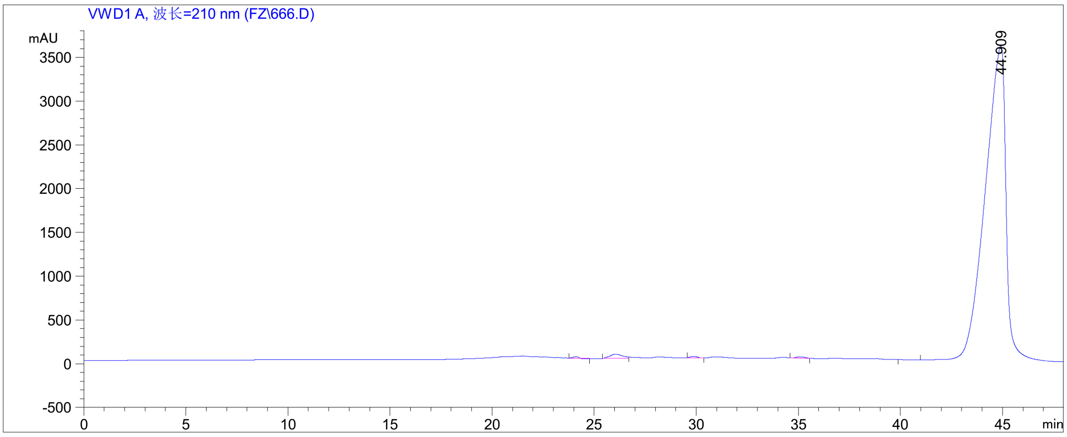

Peak	Retention time	Peak type	Peak width	Peak area	Peak height	Peak area%
1	44.909	BBA	1.0625	2.60085e5	3607.31494	100.0000

Compound 9

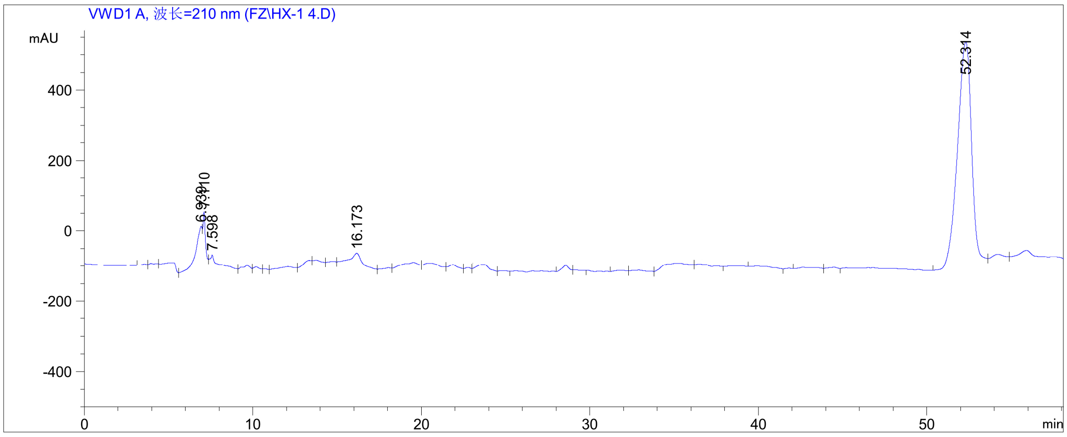

Peak #	Retention time [min]	Peak type	Peak width [min]	Peak area mAU *s	Peak height [mAU]	Peak area% %
1	6.939	VV	0.3935	3479.65039	132.59836	4.0310
2	7.110	VV	0.1749	2167.83740	173.49944	2.5113
3	7.598	VV	0.6026	2520.67188	50.76414	2.9201
4	16.173	VV	1.0148	4370.81543	54.98335	5.0633
5	52.314	BV	0.9017	7.37833e4	648.77490	85.4743

Table S1. Putative annotation of metabolites produced in the non-cultures of *Phomopsis asparagi* DHS-48 and *Phomopsis* sp. DHS-11, and their co-culture. Annotation/identification was by GNPS, based on the *m/z* [M+H]+ or other adducts (specified), predicted molecular formula, fragmentation pattern and spectral data analysis. The source of the compound is indicated as A - co-culture, B - *Phomopsis asparagi* and C - *Phomopsis* sp.

No.	Compound Hits in Library	Structure	Molecular formula (m/z)	Parent mass m/z [M+H]+	MS/MS fragmentation	Source	Libraries & IDs
A-1	Sylvestroside I	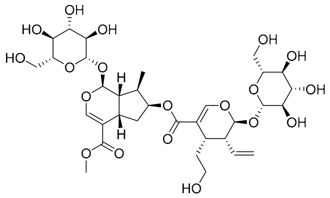	$C_{33}H_{48}O_{19}$ $[M+H]^+$	748.863	230.7750, 299.1690, 556.0440, 719.5250	A	GNPS, CCMSLIB00006469379 Dictionary of Natural Products CAS Number: 71431-22-6
A-2	5-hydroxy-3-(4-hydroxyphenyl)-10-(2,3,4-trifluorophenyl)-9,10-dihydropyrano[2,3-f]chromene-4,8-dione	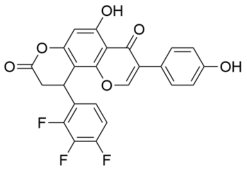	$C_{24}H_{13}F_3O_6$ Na $[M+Na]^+$	477.696	313.0430, 375.1210, 459.0800, 476.3120	C	GNPS, CCMSLIB00006428870 CAS Number: 1574399-17-9

A-3	Guaiacin	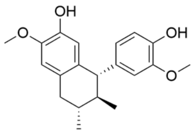	$C_{20}H_{24}O_4Na$ [M+Na]$^+$	350.507	336.1250, 349.1440, 350.9870, 351.1470	B	GNPS, CCMSLIB00006443332 Dictionary of Natural Products CAS Number: 36531-08-5
A-4	2-(3,4-dihydroxyphenyl) -3,5-dihydroxy-10-(4-(octyloxy)phenyl) -9,10-dihydropyrano[2,3-f]chromene-4,8-dione	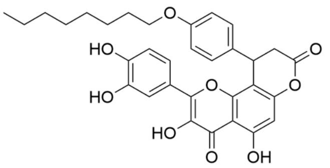	$C_{32}H_{32}O_9Na$ [M+Na]$^+$	582.485	375.1330, 419.1100, 493.1840, 582.8250	A	GNPS, CCMSLIB00006428119 CAS Number: 1574338-01-4
A-5	Phytosphingosine	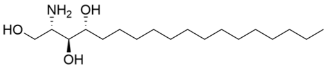	$C_{18}H_{40}NO_3$ [M+H]$^+$	318.537	264.2682, 265.2520, 270.2788, 282.2783, 300.2889	B	GNPS, CCMSLIB00003136666 Natural Product Atlas, NPA004335 CAS Number: 554-62-1
A-6	3-Hydroxyoctadecanoic Acid	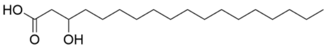	$C_{18}H_{35}O_2$ [M+H-H2O]$^+$	284.22	185.1141, 199.1053, 213.1945 , 227.1921 , 247.1897	A, B, C	GNPS, CCMSLIB00003136272 Natural Product Atlas, NPA005900 CAS Number: 17773-30-7
A-7	*meso*-Zeaxanthin	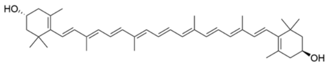	$C_{40}H_{56}O_2Na$ [M+Na]$^+$	591.161	495.3130 , 555.2940 , 573.3060 , 591.4330	B	GNPS, CCMSLIB00006383119 PMID:12189420 CAS Number: 31272-50-1
A-8	O-succinyl-L-homoserine	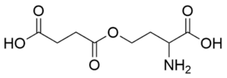	$C_8H_{13}NO_6Na$ [M+Na]$^+$	241.836	197.0420, 224.0530	B	GNPS, CCMSLIB00010103277 CAS Number: 1492-23-5
A-9	Cytosine	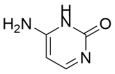	$C_4H_6N_3O$ [M+H]$^+$	112.838	95.0241, 112.0506	B	GNPS, CCMSLIB00005883678 CAS Number: 71-30-7
A-10	Methyl-1,4-benzoquinone	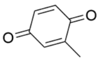	$C_7H_7O_2$ [M+H]$^+$	123.829	122.9610, 123.0800	A, B, C	GNPS, CCMSLIB00006447359 CAS Number: 553-97-9
A-11	Karacoline	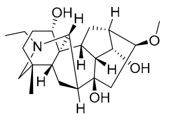	$C_{22}H_{36}NO_4$ [M+H]$^+$	379.055	378.2100, 379.2140	C	GNPS, CCMSLIB00006499319 CAS Number: 39089-30-0
A-12	Ajugasterone C	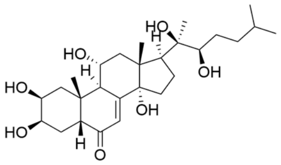	$C_{27}H_{44}O_7Na$ [M+Na]$^+$	503.012	503.2860, 503.3520	A	GNPS, CCMSLIB00006465857 CAS Number: 23044-80-6
A-13	N-Fructosyl isoleucine	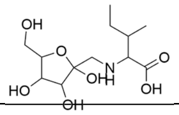	$C_{12}H_{22}NO_6$ [M+H-H2O]$^+$	275.79	212.1283, 230.1376, 258.1294,	C	GNPS, CCMSLIB00005743748

					276.1454		
B-14	4-(5-(7-chloro-5'-hydroxy-4,6-dimethoxy-3'-(methoxycarbonyl)-2',7'-dimethyl-3-oxo-7',8'-dihydro-3H,4'H-spiro[benzofuran-2,6'-quinolin]-4'-yl)furan-2-yl)benzoic acid	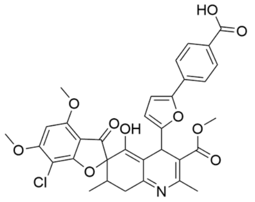	$C_{33}H_{28}ClNO_{10}Na$ [M+Na]$^+$	657.006	655.2700, 655.6950, 656.1270, 656.2670	B, C	GNPS, CCMSLIB00006452823
B-15	Hetisine	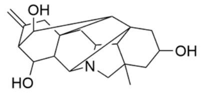	C20H28NO3 [M+H]$^+$	330.779	312.1900, 330.1700	A, C	GNPS, CCMSLIB00006570203
B-16	Oxybutynin|4-(diethylamino)but-2-ynyl 2-cyclohexyl-2-hydroxy-2-phenylacetate	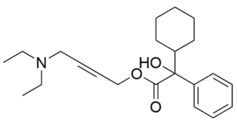	$C_{22}H_{34}NO_3$ [M+H]$^+$	358.902	124.1110, 142.1214, 340.2264, 358.2378	A	GNPS, CCMSLIB00005773582
B-17	Dorrestein	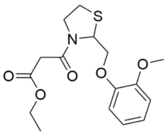	$C_{16}H_{21}NO_5SNa$ [M+Na]$^+$	362.936	238.0493, 362.1053	C	GNPS, CCMSLIB00000078575
B-18	(*S*)-methyl 2-(2-(3-isopropyl-4-oxo-3,4-dihydrophthalazin-1-yl)acetamido)-4-(methylthio)butanoate	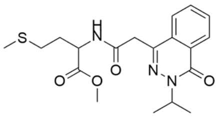	$C_{18}H_{26}N_3O_4S$ [M+H]$^+$	393.062	392.1610, 393.1640	B	GNPS, CCMSLIB00006532114
B-19	Raloxifene	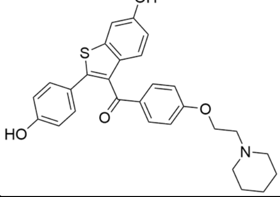	$C_{28}H_{28}NO_4S$ [M+H]$^+$	475.01	269.0270, 474.1749	A, B, C	GNPS, CCMSLIB00006115453 CAS Number: 84449-90-1
B-20	Nodakenin	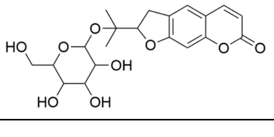	$C_{20}H_{24}O_9Na$ [M+Na]$^+$	430,976	413.2520, 431.0950, 431.1610	C	GNPS, CCMSLIB00006471515

B-21	7-hydroxy-3-(4-hydroxyphenyl)-4H-chromen-4-one	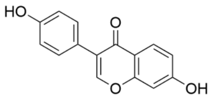	$C_{15}H_{11}O_4$ $[M+H]^+$	255.959	199.0830, 255.0700, 256.0770	A, B, C	GNPS, CCMSLIB00006575950
B-22	Etoposide	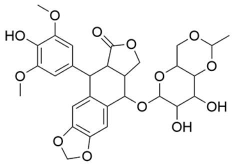	$C_{29}H_{33}O_{13}$ $[M+H]^+$	588.968	557.1990, 588.4100, 588.9120, 589.1690	A	GNPS, CCMSLIB00006445012
B-23	Dopamine-C22:5	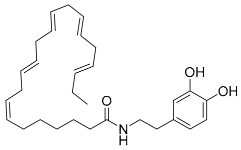	$C_{30}H_{44}NO_3$ $[M+H]^+$	467.332	154.0846, 334.2428, 353.2465, 466.3301	C	GNPS, CCMSLIB00010011797
B-24	N-(1,3-dihydroxy-2-methylpropan-2-yl)-3-methyl-4-oxo-2-phenyl-4H-chromene-8-carboxamide	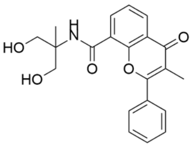	$C_{21}H_{22}NO_5$ $[M+H]^+$	368.988	368.1540, 368.1990, 369.1550	A, C	GNPS, CCMSLIB00006484105
B-25	2,3-dihydroxypropyl stearate	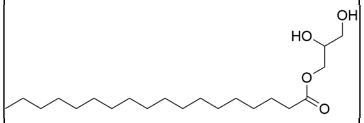	$C_{21}H_{42}O_4Na$ $[M+Na]^+$	380.866	197.0820, 216.0910, 238.0720, 381.0700	C	GNPS, CCMSLIB00006412457
B-26	purpurin	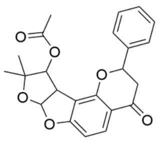	$C_{23}H_{23}O_6$ $[M+H]^+$	395.073	277.5460, 377.1430, 382.1630, 395.1940	C	GNPS, CCMSLIB00006391266 CAS Number: 75775-33-6
B-27	Asperuloside	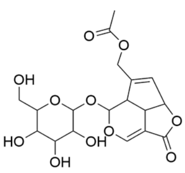	$C_{18}H_{22}O_{11}Na$ $[M+Na]^+$	436.827	333.0850, 351.1240, 356.0950, 411.1270, 437.1080	B	GNPS, CCMSLIB00006422914 CAS Number: 14259-45-1
B-28	methyl 2-ethyl-4-[(3R,4R,5S)-5-hydroxy-4,5-dimethyl-2-oxooxolan-3-yl]-2-methyl-3-oxobutanoate	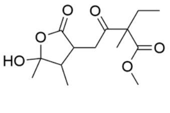	$C_{14}H_{22}O_6Na$ $[M+Na]^+$	309.953	291.1205, 309.1309	C	GNPS, CCMSLIB00004714908 CAS Number: 129514-40-5
C-29	Docosanol	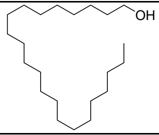	$C_{22}H_{45}O$ $[M-H]^-$	325.19	79.9559, 119.0490, 170.0034,	B	GNPS, CCMSLIB00004702727

					183.0114, 197.1430, 325.1842		
C-30	9-Hydroxy-10E,12Z-octadecadienoic acid	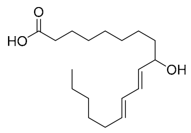	$C_{18}H_{31}O_3$ [M-H]$^-$	295.417	97.1558, 171.1534, 195.1563, 251.4050, 277.3235	A, B	GNPS, CCMSLIB00003136732
D-31	Beauvericin	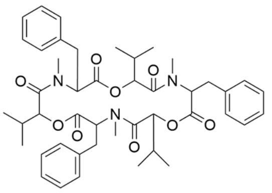	$C_{45}H_{57}N_3O_9$ Na [M+Na]$^+$	806.395	384.1761, 545.2592, 645.3112	A	GNPS, CCMSLIB00005723573 Natural Product Atlas, NPA001888
E-32	2(3H)-Furanone, 5-[4-[1-(acetyloxy)-4-oxo-5-(2-penten-1-yl)-2-cyclopenten-1-yl]butyl]dihydro-(ACI)	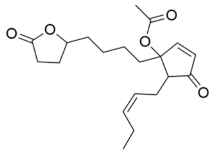	$C_{20}H_{28}O_5Na$ [M+Na]$^+$	371.034	311.1626, 372.1873	B	GNPS, CCMSLIB00000852834
F-33	Chlordiazepoxide	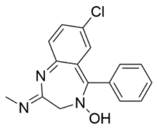	$C_{16}H_{13}ClN_3$ O [M-H]$^-$	297.421	221.0719, 241.0300, 249.0669, 253.0538, 254.0615	A	GNPS, CCMSLIB00005733539 CAS Number: 58-25-3
G-34	Hydroxygardnutine	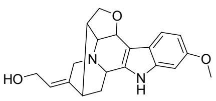	$C_{21}H_{23}N_2O_5$ [M+COOH]$^-$	383.533	337.1543, 338.1487, 338.1639, 339.1542	A, B, C	GNPS, CCMSLIB00005744488
H-35	Undecylprodigiosin	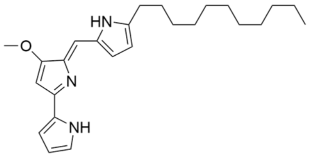	$C_{25}H_{36}N_3O$ [M+H]$^+$	393.307	238.0968, 239.1039, 379.2613, 394.2581	A	GNPS, CCMSLIB00005724040
I-36	Aconitine	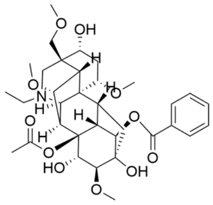	$C_{34}H_{48}NO_{11}$ [M+H]$^+$	646.997	646.3810, 647.3810, 647.4010	A	GNPS, CCMSLIB00006507956
J-37	2-((11aS)-5-(4-fluorophenyl)-1,3-dioxo-11,11a-dihydro-1H-imidazo[1',5':1,6]	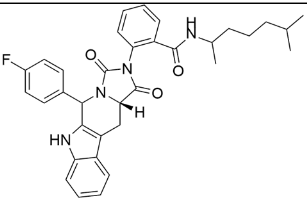	$C_{34}H_{35}FN_4O_3Na$ [M+Na]$^+$	590.056	589.2600, 589.4220, 590.2530, 590.2800, 591.2630	B	GNPS, CCMSLIB00006536412 CAS Number: 956918-50-6

	pyrido[3,4-b]indol-2(3H,5H,6H)-yl)-N-(6-methylheptan-2-yl)benzamide						
J-38	Spiro[2H-furo[2,3-e]isoindole-2,1′(2′H)-naphthalene]-7(3H)-butanoic acid, 3′,4′,4′a,5′,6,6′,7′,8,8′,8′a-decahydro-4,6′,7′-trihydroxy-2′,5′,5′,8′a-tetramethyl-6-oxo-, (1′R,2′R,4′aS,6′S,7′R,8′aS)- (9CI, ACI)	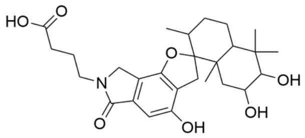	$C_{27}H_{37}NO_7Na$ $[M+Na]^+$	511.127	510.0832, 511.0973, 511.2525	B	GNPS, CCMSLIB00000851579 Natural Product Atlas, NPA008246

Table S2. Gibbs free energies[a] and equilibrium populations[b] of low-energy conformers of phomoxanthone L (**1**)

Conformers	In MeOH	
	G^a	P (%)^b
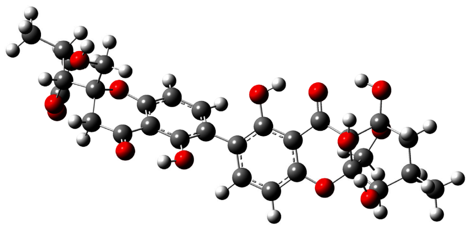 **1-1**	-1342070.06533743	59.01
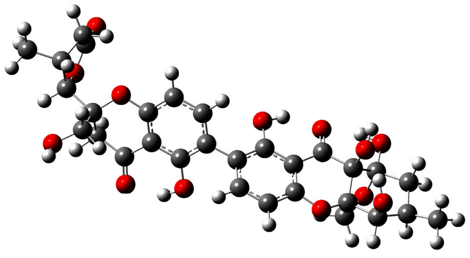 **1-2**	-1342069.80241074	37.85

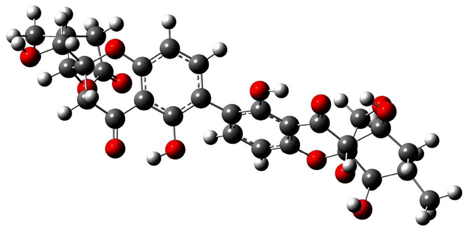 1-3	-1342068.22798815	2.65
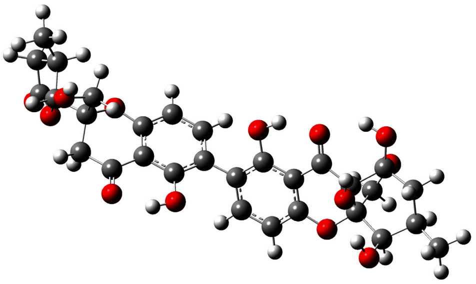 1-4	-1342067.2271097	0.49

[a]B3LYP/6-31G(d,p), in kcal/mol. [b]From *G* values at 298.15K.

Table S3. Cartesian coordinates for the low-energy reoptimized MMFF conformers of phomoxanthone L (**1**) at B3LYP/6-31G(d,p) level of theory in gas

1-1		Standard Orientation (Ångstroms)			
Center number	Atomic number	Atomic Type	X	Y	Z
1.	6.	0.	-0.558556	0.514664	-0.469617
2.	6.	0.	-1.044212	1.653112	-1.133430
3.	6.	0.	-2.398926	1.932471	-1.294050
4.	6.	0.	-3.339299	1.054799	-0.768878
5.	6.	0.	-2.915451	-0.104713	-0.064043
6.	6.	0.	-1.513062	-0.367952	0.070852
7.	8.	0.	-4.644949	1.356064	-0.965429
8.	6.	0.	-5.634940	0.372300	-0.660086
9.	6.	0.	-5.341927	-0.487448	0.572878
10.	6.	0.	-3.881589	-0.962663	0.564517
11.	6.	0.	-6.994411	1.103268	-0.547839
12.	6.	0.	-8.147427	0.061522	-0.427622
13.	6.	0.	-7.801324	-1.088778	0.537747
14.	8.	0.	-3.578748	-2.009008	1.183481
15.	8.	0.	-1.101685	-1.459152	0.731909
16.	6.	0.	-9.469939	0.741062	-0.062260
17.	8.	0.	-6.989477	2.083641	0.469311
18.	6.	0.	-5.685819	-0.746861	-1.737860
19.	8.	0.	-6.151816	-1.927626	-1.069830
20.	6.	0.	-6.378262	-1.619051	0.325486
21.	8.	0.	-5.536982	0.186920	1.813695
22.	8.	0.	-6.211487	-2.736657	1.118328

23.	6.	0.	2.802805	-1.301514	-0.497393
24.	6.	0.	3.679109	-0.289418	-0.127242
25.	6.	0.	3.196492	1.013947	0.128034
26.	6.	0.	1.798710	1.276323	0.027632
27.	6.	0.	0.899249	0.253687	-0.346356
28.	6.	0.	1.442301	-1.012963	-0.602855
29.	8.	0.	5.003693	-0.607077	-0.035368
30.	6.	0.	5.896052	0.279101	0.680847
31.	6.	0.	5.598894	1.748744	0.355029
32.	6.	0.	4.127661	2.085025	0.436880
33.	8.	0.	3.758586	3.241036	0.702108
34.	8.	0.	1.336359	2.506676	0.283599
35.	6.	0.	5.714811	-0.005217	2.187308
36.	8.	0.	6.683796	0.757763	2.892610
37.	6.	0.	7.323255	-0.102107	0.234722
38.	6.	0.	7.656287	-1.621623	0.248429
39.	6.	0.	7.475676	-2.019103	-1.224378
40.	6.	0.	7.588773	-0.716812	-1.999116
41.	8.	0.	7.540647	0.335395	-1.121466
42.	8.	0.	7.707179	-0.550411	-3.182809
43.	6.	0.	9.079791	-1.871426	0.761502
44.	1.	0.	8.013977	0.462284	0.865506
45.	1.	0.	6.947214	-2.161659	0.880041
46.	1.	0.	-0.322058	2.347184	-1.546572
47.	1.	0.	-2.736465	2.812025	-1.828858
48.	1.	0.	-7.133231	1.659092	-1.483546
49.	1.	0.	-8.265815	-0.386535	-1.421446
50.	1.	0.	-7.902240	-0.766767	1.579758
51.	1.	0.	-8.486820	-1.928952	0.392280
52.	1.	0.	-1.909634	-1.936330	1.049313
53.	1.	0.	-9.394634	1.247653	0.903032
54.	1.	0.	-10.279640	0.006127	-0.010140
55.	1.	0.	-9.744081	1.494754	-0.807543
56.	1.	0.	-6.658132	1.664294	1.278518
57.	1.	0.	-6.355516	-0.481135	-2.562741
58.	1.	0.	-4.688048	-0.927761	-2.153439
59.	1.	0.	-5.583024	-0.498100	2.499182
60.	1.	0.	-5.258039	-2.940640	1.141797
61.	1.	0.	3.183863	-2.297418	-0.690371
62.	1.	0.	0.765960	-1.811846	-0.883590
63.	1.	0.	6.154207	2.400108	1.033149
64.	1.	0.	5.930702	1.966387	-0.666786
65.	1.	0.	2.127075	3.067228	0.518002

66.	1.	0.	5.833377	-1.082403	2.367443
67.	1.	0.	4.690927	0.265696	2.478290
68.	1.	0.	6.461779	0.746094	3.831563
69.	1.	0.	8.220976	-2.730215	-1.589283
70.	1.	0.	6.485229	-2.438501	-1.422828
71.	1.	0.	9.817290	-1.334928	0.153743
72.	1.	0.	9.188896	-1.533389	1.797024
73.	1.	0.	9.326633	-2.937097	0.724349

**1-2**		Standard Orientation (Ångstroms)			
Center number	Atom number	Type	X	Y	Z
1.	6.	0.	-0.565846	-0.710699	-0.316862
2.	6.	0.	-1.074142	-2.018264	-0.383030
3.	6.	0.	-2.432354	-2.321518	-0.340925
4.	6.	0.	-3.354460	-1.288213	-0.232555
5.	6.	0.	-2.910029	0.061024	-0.187182
6.	6.	0.	-1.503620	0.335826	-0.216836
7.	8.	0.	-4.663558	-1.630444	-0.174247
8.	6.	0.	-5.619879	-0.634891	0.192834
9.	6.	0.	-5.342186	0.768254	-0.354456
10.	6.	0.	-3.863413	1.137003	-0.161755
11.	6.	0.	-7.013978	-1.156860	-0.228639
12.	6.	0.	-8.123020	-0.220443	0.336355
13.	6.	0.	-7.772018	1.269880	0.166122
14.	8.	0.	-3.541039	2.344901	-0.090027
15.	8.	0.	-1.072669	1.603073	-0.154637
16.	6.	0.	-9.491385	-0.562174	-0.259631
17.	8.	0.	-7.099647	-1.381951	-1.621104
18.	6.	0.	-5.574009	-0.321330	1.715544
19.	8.	0.	-6.013237	1.037228	1.858408
20.	6.	0.	-6.317784	1.572250	0.549419
21.	8.	0.	-5.617181	0.918429	-1.745819
22.	8.	0.	-6.139215	2.940560	0.513505
23.	6.	0.	2.816217	0.837369	-1.199778
24.	6.	0.	3.690543	0.064030	-0.447270
25.	6.	0.	3.196225	-0.968867	0.380999
26.	6.	0.	1.794430	-1.225817	0.420661
27.	6.	0.	0.896848	-0.449089	-0.345881
28.	6.	0.	1.448357	0.572305	-1.132317

29.	8.	0.	5.022420	0.350456	-0.538534
30.	6.	0.	5.988589	-0.623522	-0.077173
31.	6.	0.	5.547517	-1.255484	1.249064
32.	6.	0.	4.106557	-1.711304	1.235312
33.	8.	0.	3.732158	-2.640539	1.969906
34.	8.	0.	1.325064	-2.204436	1.205176
35.	6.	0.	6.129702	-1.689812	-1.184245
36.	8.	0.	7.172185	-2.576393	-0.802872
37.	6.	0.	7.318123	0.141202	0.096219
38.	6.	0.	7.727429	1.071187	-1.082201
39.	6.	0.	7.275539	2.454139	-0.589637
40.	6.	0.	7.176213	2.318142	0.920554
41.	8.	0.	7.243886	0.990753	1.258090
42.	8.	0.	7.059580	3.178317	1.750577
43.	6.	0.	9.236541	0.997208	-1.344785
44.	1.	0.	8.088253	-0.606859	0.298744
45.	1.	0.	7.189723	0.793918	-1.991805
46.	1.	0.	-0.366718	-2.835728	-0.453453
47.	1.	0.	-2.786040	-3.344788	-0.378848
48.	1.	0.	-7.134565	-2.145432	0.231882
49.	1.	0.	-8.171868	-0.410914	1.415053
50.	1.	0.	-7.936670	1.594188	-0.867176
51.	1.	0.	-8.410917	1.888721	0.803116
52.	1.	0.	-1.869747	2.186723	-0.084166
53.	1.	0.	-9.484618	-0.441252	-1.345631
54.	1.	0.	-10.267730	0.085251	0.160592
55.	1.	0.	-9.765240	-1.600732	-0.047023
56.	1.	0.	-6.782302	-0.580783	-2.066270
57.	1.	0.	-6.221267	-0.997449	2.284266
58.	1.	0.	-4.553356	-0.421048	2.101227
59.	1.	0.	-5.679927	1.871811	-1.914971
60.	1.	0.	-5.180423	3.113148	0.560346
61.	1.	0.	3.206309	1.624246	-1.834566
62.	1.	0.	0.775674	1.182868	-1.722665
63.	1.	0.	6.190309	-2.104727	1.490254
64.	1.	0.	5.650850	-0.517429	2.052872
65.	1.	0.	2.112589	-2.599351	1.673156
66.	1.	0.	6.346475	-1.190989	-2.138810
67.	1.	0.	5.169891	-2.212246	-1.295944
68.	1.	0.	7.152485	-3.345012	-1.385908
69.	1.	0.	7.962751	3.265346	-0.842277
70.	1.	0.	6.283799	2.723077	-0.964305
71.	1.	0.	9.805813	1.260550	-0.446095

72.	1.	0.	9.533460	-0.012711	-1.645358
73.	1.	0.	9.528779	1.688777	-2.141179

**1-3**		Standard Orientation (Ångstroms)			
Center number	Atom number	Type	X	Y	Z
1.	6.	0.	-0.296059	-0.272941	-0.009980
2.	6.	0.	-0.642981	-1.633528	0.029197
3.	6.	0.	-1.957191	-2.092562	0.077254
4.	6.	0.	-2.998216	-1.172842	0.076987
5.	6.	0.	-2.717029	0.219887	0.022837
6.	6.	0.	-1.352480	0.657664	-0.010177
7.	8.	0.	-4.259601	-1.664383	0.133859
8.	6.	0.	-5.343286	-0.764342	0.369563
9.	6.	0.	-5.203175	0.613010	-0.285032
10.	6.	0.	-3.788372	1.171102	-0.074876
11.	6.	0.	-6.644241	-1.481801	-0.064712
12.	6.	0.	-7.883246	-0.639454	0.364778
13.	6.	0.	-7.694660	0.865216	0.088448
14.	8.	0.	-3.614038	2.411886	-0.103683
15.	8.	0.	-1.077067	1.968701	-0.060313
16.	6.	0.	-9.167529	-1.183124	-0.267346
17.	8.	0.	-6.627953	-1.832612	-1.432922
18.	6.	0.	-5.414043	-0.326927	1.859750
19.	8.	0.	-6.013879	0.976071	1.868849
20.	6.	0.	-6.307555	1.366506	0.506936
21.	8.	0.	-5.426912	0.613402	-1.693423
22.	8.	0.	-6.283594	2.739419	0.364686
23.	6.	0.	2.926905	1.639869	0.734611
24.	6.	0.	3.831684	1.015619	-0.115167
25.	6.	0.	3.408822	-0.030172	-0.962678
26.	6.	0.	2.052431	-0.461227	-0.921064
27.	6.	0.	1.123364	0.163694	-0.057815
28.	6.	0.	1.599582	1.208406	0.744410
29.	8.	0.	5.120450	1.472016	-0.091815
30.	6.	0.	6.181869	0.709896	-0.712981
31.	6.	0.	5.706400	0.027232	-2.005103
32.	6.	0.	4.343416	-0.624498	-1.901188
33.	8.	0.	4.037930	-1.553840	-2.662375
34.	8.	0.	1.648749	-1.455351	-1.720793

35.	6.	0.	7.234128	1.786145	-1.032772
36.	8.	0.	8.346738	1.144502	-1.636650
37.	6.	0.	6.774743	-0.310077	0.286124
38.	6.	0.	6.989433	0.207501	1.737992
39.	6.	0.	5.829731	-0.441209	2.508822
40.	6.	0.	5.373049	-1.601456	1.639973
41.	8.	0.	5.920009	-1.467926	0.390020
42.	8.	0.	4.645513	-2.514486	1.921345
43.	6.	0.	8.365200	-0.200968	2.278442
44.	1.	0.	7.715350	-0.656450	-0.151308
45.	1.	0.	6.899713	1.295916	1.763643
46.	1.	0.	0.158581	-2.362526	0.025656
47.	1.	0.	-2.185414	-3.150604	0.122537
48.	1.	0.	-6.675055	-2.435189	0.477520
49.	1.	0.	-7.973973	-0.751841	1.451929
50.	1.	0.	-7.839186	1.086387	-0.974540
51.	1.	0.	-8.433787	1.451148	0.642922
52.	1.	0.	-1.940698	2.453497	-0.074618
53.	1.	0.	-9.113760	-1.142114	-1.358072
54.	1.	0.	-10.035160	-0.602873	0.062932
55.	1.	0.	-9.332391	-2.228405	0.013590
56.	1.	0.	-6.383591	-1.039635	-1.935030
57.	1.	0.	-6.006641	-1.028230	2.456879
58.	1.	0.	-4.409333	-0.273056	2.294137
59.	1.	0.	-5.566971	1.537542	-1.953280
60.	1.	0.	-5.352340	3.023509	0.424332
61.	1.	0.	3.262454	2.446702	1.375873
62.	1.	0.	0.901417	1.703594	1.408791
63.	1.	0.	5.624300	0.783093	-2.797866
64.	1.	0.	6.433317	-0.711098	-2.346615
65.	1.	0.	2.444965	-1.744515	-2.245549
66.	1.	0.	7.514450	2.310128	-0.107994
67.	1.	0.	6.764631	2.521817	-1.700524
68.	1.	0.	8.982728	1.819866	-1.902081
69.	1.	0.	6.102815	-0.807200	3.501446
70.	1.	0.	4.975874	0.233608	2.628730
71.	1.	0.	8.486102	-1.290212	2.267619
72.	1.	0.	9.169464	0.229857	1.673231
73.	1.	0.	8.497216	0.140376	3.310022

**1-4**	Standard Orientation<br>(Ångstroms)

Center number	Atom number	Type	X	Y	Z
1.	6.	0.	-0.505109	-0.761861	-0.303708
2.	6.	0.	-1.032043	-2.063439	-0.316112
3.	6.	0.	-2.394549	-2.346080	-0.254272
4.	6.	0.	-3.301757	-1.297306	-0.181384
5.	6.	0.	-2.838438	0.046710	-0.189627
6.	6.	0.	-1.428250	0.300333	-0.237586
7.	8.	0.	-4.616074	-1.618662	-0.104658
8.	6.	0.	-5.555284	-0.596903	0.231223
9.	6.	0.	-5.261338	0.780990	-0.369038
10.	6.	0.	-3.776197	1.135687	-0.199872
11.	6.	0.	-6.959837	-1.113379	-0.161878
12.	6.	0.	-8.051678	-0.140886	0.374774
13.	6.	0.	-7.680190	1.336681	0.148113
14.	8.	0.	-3.438021	2.341558	-0.176917
15.	8.	0.	-0.979915	1.563117	-0.225752
16.	6.	0.	-9.428453	-0.483821	-0.200942
17.	8.	0.	-7.058698	-1.387423	-1.544809
18.	6.	0.	-5.494542	-0.227974	1.740896
19.	8.	0.	-5.913819	1.141164	1.836642
20.	6.	0.	-6.219110	1.631859	0.510495
21.	8.	0.	-5.544529	0.884095	-1.763244
22.	8.	0.	-6.020869	2.995218	0.422643
23.	6.	0.	2.889281	0.699802	-1.275019
24.	6.	0.	3.757729	-0.052892	-0.494792
25.	6.	0.	3.256230	-1.038761	0.379067
26.	6.	0.	1.855011	-1.279896	0.435015
27.	6.	0.	0.960590	-0.521141	-0.353468
28.	6.	0.	1.518675	0.457671	-1.187401
29.	8.	0.	5.091048	0.226217	-0.604188
30.	6.	0.	6.083334	-0.676967	-0.064621
31.	6.	0.	5.606648	-1.313193	1.251554
32.	6.	0.	4.167008	-1.778211	1.231969
33.	8.	0.	3.796596	-2.685026	1.993418
34.	8.	0.	1.379124	-2.227352	1.255536
35.	6.	0.	6.421986	-1.747793	-1.115164
36.	8.	0.	5.343880	-2.661083	-1.199812
37.	6.	0.	7.335108	0.201660	0.181808
38.	6.	0.	7.769930	1.120431	-0.998797
39.	6.	0.	7.159721	2.474968	-0.608180
40.	6.	0.	6.948993	2.387792	0.893410
41.	8.	0.	7.099867	1.082069	1.293409

42.	8.	0.	6.696915	3.262735	1.675144
43.	6.	0.	9.297567	1.177965	-1.125270
44.	1.	0.	8.149984	-0.464995	0.484594
45.	1.	0.	7.341879	0.763335	-1.938055
46.	1.	0.	-0.335661	-2.892201	-0.358787
47.	1.	0.	-2.762037	-3.365215	-0.250805
48.	1.	0.	-7.091358	-2.082693	0.335162
49.	1.	0.	-8.097256	-0.291418	1.459937
50.	1.	0.	-7.847316	1.625818	-0.895249
51.	1.	0.	-8.305623	1.987673	0.766211
52.	1.	0.	-1.768342	2.159786	-0.173443
53.	1.	0.	-9.426311	-0.402217	-1.290629
54.	1.	0.	-10.192930	0.189560	0.199895
55.	1.	0.	-9.715938	-1.509995	0.050447
56.	1.	0.	-6.731131	-0.608030	-2.020296
57.	1.	0.	-6.146976	-0.873352	2.338695
58.	1.	0.	-4.472453	-0.327621	2.122600
59.	1.	0.	-5.590485	1.831535	-1.967353
60.	1.	0.	-5.058814	3.153876	0.452748
61.	1.	0.	3.285424	1.452662	-1.946382
62.	1.	0.	0.849924	1.051952	-1.798563
63.	1.	0.	6.243027	-2.156599	1.530710
64.	1.	0.	5.681151	-0.564704	2.048858
65.	1.	0.	2.167187	-2.628142	1.715731
66.	1.	0.	7.352472	-2.251882	-0.806513
67.	1.	0.	6.605226	-1.256612	-2.080327
68.	1.	0.	5.531403	-3.300732	-1.897696
69.	1.	0.	7.792592	3.334330	-0.842550
70.	1.	0.	6.182811	2.636478	-1.071924
71.	1.	0.	9.757843	1.517973	-0.190741
72.	1.	0.	9.715200	0.194444	-1.365601
73.	1.	0.	9.596253	1.871674	-1.917116

Table S4. Gibbs free energies[a] and equilibrium populations[b] of low-energy conformers of phomoxanthone M (**2**)

Conformers	In MeOH	
	G^a	*P* (%)^b
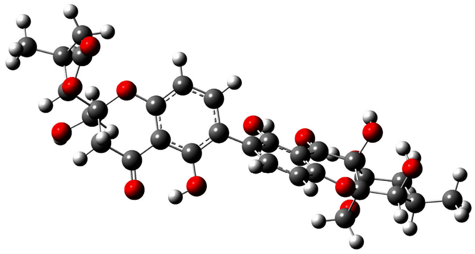	-1342069.61980533	29.44

2-1		
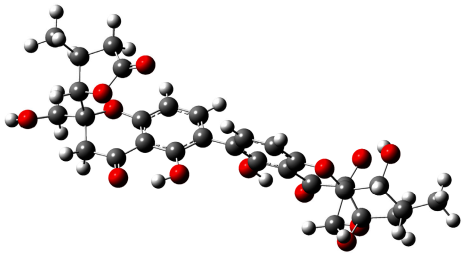 2-2	-1342069.89026214	46.49
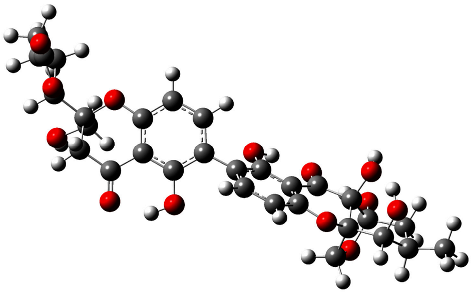 2-3	-1342067.9173707	1.66
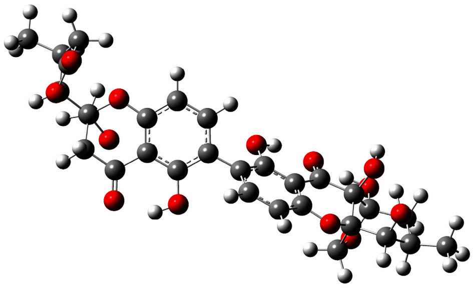 2-4	-1342069.45853526	22.42

[a]B3LYP/6-31G(d,p), in kcal/mol. [b]From *G* values at 298.15K.

Table S5. Cartesian coordinates for the low-energy reoptimized MMFF conformers of phomoxanthone M (**2**) at B3LYP/6-31G(d,p) level of theory in gas

**2-1**		Standard Orientation (Ångstroms)			
Center number	Atomic number	Atomic Type	X	Y	Z
1.	6.	0.	0.559666	0.585643	-0.412052
2.	6.	0.	1.048955	1.830053	-0.842183
3.	6.	0.	2.403934	2.142166	-0.915966
4.	6.	0.	3.341653	1.182553	-0.555320
5.	6.	0.	2.915185	-0.106105	-0.133310
6.	6.	0.	1.512120	-0.386731	-0.051463
7.	8.	0.	4.647204	1.535543	-0.625396
8.	6.	0.	5.626907	0.677866	-0.037980
9.	6.	0.	5.348680	-0.821797	-0.178090
10.	6.	0.	3.881363	-1.129738	0.156237
11.	6.	0.	7.000439	1.070712	-0.632822
12.	6.	0.	8.139380	0.321304	0.120953

13.	6.	0.	7.799159	-1.160539	0.372067
14.	8.	0.	3.575909	-2.273624	0.565585
15.	8.	0.	1.099157	-1.591554	0.366990
16.	6.	0.	9.481184	0.489827	-0.597130
17.	8.	0.	7.035135	0.915488	-2.036746
18.	6.	0.	5.635054	0.785215	1.513187
19.	8.	0.	6.097835	-0.482636	2.000735
20.	6.	0.	6.362765	-1.350647	0.873990
21.	8.	0.	5.579649	-1.339033	-1.486899
22.	8.	0.	6.196902	-2.678142	1.214423
23.	6.	0.	-2.807258	-1.187120	-0.766827
24.	6.	0.	-3.679350	-0.260266	-0.210735
25.	6.	0.	-3.193407	0.974946	0.274262
26.	6.	0.	-1.795514	1.249133	0.217888
27.	6.	0.	-0.899160	0.310600	-0.339914
28.	6.	0.	-1.446753	-0.885502	-0.824703
29.	8.	0.	-5.003304	-0.590147	-0.165795
30.	6.	0.	-5.886171	0.150987	0.709458
31.	6.	0.	-5.593961	1.655692	0.643929
32.	6.	0.	-4.121890	1.973216	0.775940
33.	8.	0.	-3.749922	3.062365	1.243343
34.	8.	0.	-1.330500	2.411482	0.693823
35.	6.	0.	-5.681453	-0.394250	2.138763
36.	8.	0.	-6.642587	0.225839	2.981440
37.	6.	0.	-7.320190	-0.150544	0.224935
38.	6.	0.	-7.651068	-1.649239	-0.031145
39.	6.	0.	-7.484990	-1.772980	-1.553186
40.	6.	0.	-7.621906	-0.353716	-2.078320
41.	8.	0.	-7.563182	0.523176	-1.025867
42.	8.	0.	-7.764076	0.022679	-3.210091
43.	6.	0.	-9.069306	-1.990073	0.442692
44.	1.	0.	-8.000409	0.287220	0.959134
45.	1.	0.	-6.935292	-2.292096	0.486280
46.	1.	0.	0.328927	2.591908	-1.116005
47.	1.	0.	2.743454	3.118675	-1.239619
48.	1.	0.	7.125160	2.146623	-0.457664
49.	1.	0.	8.226125	0.795899	1.105631
50.	1.	0.	7.932811	-1.750951	-0.540826
51.	1.	0.	8.466530	-1.583116	1.129036
52.	1.	0.	1.906537	-2.128038	0.570983
53.	1.	0.	9.436915	0.077227	-1.608034
54.	1.	0.	9.749609	1.547496	-0.686159
55.	1.	0.	10.279770	-0.017102	-0.046005

56.	1.	0.	6.713336	0.023245	-2.239275
57.	1.	0.	6.290833	1.592402	1.856405
58.	1.	0.	4.626156	0.980407	1.893789
59.	1.	0.	5.647941	-2.302711	-1.397048
60.	1.	0.	5.241218	-2.831853	1.334884
61.	1.	0.	-3.191305	-2.130144	-1.137618
62.	1.	0.	-0.773976	-1.619254	-1.252406
63.	1.	0.	-5.931820	2.047800	-0.322397
64.	1.	0.	-6.145639	2.177621	1.428628
65.	1.	0.	-2.120425	2.921270	1.026690
66.	1.	0.	-4.654405	-0.171476	2.457758
67.	1.	0.	-5.793046	-1.487074	2.126743
68.	1.	0.	-6.404037	0.053449	3.900361
69.	1.	0.	-6.491689	-2.135393	-1.832771
70.	1.	0.	-8.226028	-2.416933	-2.033054
71.	1.	0.	-9.316994	-3.031904	0.216277
72.	1.	0.	-9.813043	-1.353780	-0.050271
73.	1.	0.	-9.167344	-1.844888	1.523267

**2-2**		Standard Orientation (Ångstroms)			
Center number	Atom number	Type	X	Y	Z
1.	6.	0.	0.565249	-0.685414	-0.363868
2.	6.	0.	1.075483	-1.976467	-0.576279
3.	6.	0.	2.435270	-2.275479	-0.593816
4.	6.	0.	3.356198	-1.257435	-0.379800
5.	6.	0.	2.907081	0.068771	-0.135240
6.	6.	0.	1.500503	0.344038	-0.141645
7.	8.	0.	4.667967	-1.591405	-0.421098
8.	6.	0.	5.646691	-0.550715	-0.436872
9.	6.	0.	5.308882	0.676596	0.413512
10.	6.	0.	3.850744	1.103745	0.188069
11.	6.	0.	6.999801	-1.183460	-0.030213
12.	6.	0.	8.150110	-0.151960	-0.233773
13.	6.	0.	7.769463	1.257473	0.260940
14.	8.	0.	3.526766	2.296074	0.392913
15.	8.	0.	1.065914	1.591387	0.082639
16.	6.	0.	9.455356	-0.648662	0.393940
17.	8.	0.	6.954006	-1.754286	1.260976
18.	6.	0.	5.741912	0.129181	-1.831452

19.	8.	0.	6.181032	1.473618	-1.593766
20.	6.	0.	6.355862	1.665609	-0.169802
21.	8.	0.	5.454184	0.470124	1.816393
22.	8.	0.	6.161711	2.985509	0.183771
23.	6.	0.	-2.811821	0.950044	-1.090152
24.	6.	0.	-3.688870	0.105471	-0.421919
25.	6.	0.	-3.198176	-1.007348	0.297714
26.	6.	0.	-1.797092	-1.270576	0.312379
27.	6.	0.	-0.897066	-0.421053	-0.368980
28.	6.	0.	-1.444544	0.677002	-1.047092
29.	8.	0.	-5.020150	0.401985	-0.486810
30.	6.	0.	-5.988879	-0.611365	-0.126767
31.	6.	0.	-5.551443	-1.374876	1.129666
32.	6.	0.	-4.111414	-1.830782	1.071209
33.	8.	0.	-3.740040	-2.830864	1.707472
34.	8.	0.	-1.329844	-2.325651	0.991455
35.	6.	0.	-6.129926	-1.560789	-1.335661
36.	8.	0.	-7.168117	-2.485436	-1.043551
37.	6.	0.	-7.317598	0.134734	0.119573
38.	6.	0.	-7.721466	1.177951	-0.961573
39.	6.	0.	-7.266619	2.503710	-0.333411
40.	6.	0.	-7.174976	2.218928	1.156252
41.	8.	0.	-7.245316	0.864631	1.360361
42.	8.	0.	-7.061324	2.992459	2.067946
43.	6.	0.	-9.230098	1.135901	-1.233802
44.	1.	0.	-8.089602	-0.628321	0.244966
45.	1.	0.	-7.182191	0.989875	-1.892842
46.	1.	0.	0.369170	-2.781913	-0.738463
47.	1.	0.	2.792052	-3.281628	-0.778145
48.	1.	0.	7.172250	-2.025510	-0.712194
49.	1.	0.	8.309804	-0.073831	-1.315953
50.	1.	0.	7.832933	1.317414	1.352853
51.	1.	0.	8.460376	2.003584	-0.142709
52.	1.	0.	1.860782	2.160564	0.240895
53.	1.	0.	9.341025	-0.791677	1.471283
54.	1.	0.	9.756959	-1.610094	-0.034249
55.	1.	0.	10.264130	0.068231	0.219547
56.	1.	0.	6.593221	-1.085900	1.864049
57.	1.	0.	6.444527	-0.396082	-2.487228
58.	1.	0.	4.762040	0.142345	-2.322153
59.	1.	0.	5.471809	1.348451	2.228180
60.	1.	0.	5.208360	3.174623	0.102104
61.	1.	0.	-3.199184	1.798448	-1.641936

62.	1.	0.	-0.768979	1.344428	-1.569064
63.	1.	0.	-5.655147	-0.721928	2.004004
64.	1.	0.	-6.196921	-2.242398	1.281761
65.	1.	0.	-2.117526	-2.764871	1.417292
66.	1.	0.	-5.168515	-2.065490	-1.501380
67.	1.	0.	-6.350957	-0.969193	-2.234725
68.	1.	0.	-7.147171	-3.190696	-1.701731
69.	1.	0.	-6.271779	2.803404	-0.674886
70.	1.	0.	-7.949045	3.339048	-0.508379
71.	1.	0.	-9.517791	1.905230	-1.957193
72.	1.	0.	-9.800973	1.309504	-0.314560
73.	1.	0.	-9.529667	0.162594	-1.635574

**2-3**		Standard Orientation (Ångstroms)			
Center number	Atom number	Type	X	Y	Z
1.	6.	0.	0.292763	0.238269	0.073412
2.	6.	0.	0.628580	1.481742	0.632151
3.	6.	0.	1.939879	1.916075	0.810492
4.	6.	0.	2.986412	1.090367	0.420206
5.	6.	0.	2.715497	-0.175023	-0.169119
6.	6.	0.	1.353932	-0.594255	-0.328176
7.	8.	0.	4.245570	1.546523	0.626811
8.	6.	0.	5.336677	0.634084	0.500104
9.	6.	0.	5.202312	-0.392406	-0.628614
10.	6.	0.	3.792456	-1.001670	-0.638877
11.	6.	0.	6.629677	1.473958	0.367063
12.	6.	0.	7.876251	0.542740	0.445159
13.	6.	0.	7.698838	-0.747294	-0.380864
14.	8.	0.	3.625727	-2.138389	-1.139144
15.	8.	0.	1.083702	-1.790200	-0.870071
16.	6.	0.	9.154212	1.296567	0.067152
17.	8.	0.	6.604907	2.314219	-0.768475
18.	6.	0.	5.419158	-0.336143	1.712568
19.	8.	0.	6.025716	-1.540817	1.223297
20.	6.	0.	6.316302	-1.380505	-0.184341
21.	8.	0.	5.418284	0.141879	-1.933404
22.	8.	0.	6.301694	-2.595490	-0.840085
23.	6.	0.	-2.937892	-1.098652	-1.466707
24.	6.	0.	-3.835778	-0.947537	-0.416825

25.	6.	0.	-3.402982	-0.433957	0.824067
26.	6.	0.	-2.045008	-0.037434	0.982513
27.	6.	0.	-1.124849	-0.179086	-0.080587
28.	6.	0.	-1.607644	-0.719698	-1.278446
29.	8.	0.	-5.128809	-1.326829	-0.648297
30.	6.	0.	-6.180222	-0.962968	0.277061
31.	6.	0.	-5.688278	-0.994488	1.732569
32.	6.	0.	-4.327260	-0.364493	1.941375
33.	8.	0.	-4.012750	0.088590	3.051257
34.	8.	0.	-1.629770	0.457342	2.154659
35.	6.	0.	-7.235154	-2.058492	0.043854
36.	8.	0.	-8.336979	-1.794325	0.898416
37.	6.	0.	-6.778067	0.415032	-0.091030
38.	6.	0.	-7.002113	0.677273	-1.608537
39.	6.	0.	-5.833755	1.606551	-1.971316
40.	6.	0.	-5.369210	2.194890	-0.649398
41.	8.	0.	-5.922835	1.477605	0.379761
42.	8.	0.	-4.632448	3.122639	-0.453193
43.	6.	0.	-8.370998	1.319527	-1.865207
44.	1.	0.	-7.715421	0.500592	0.465698
45.	1.	0.	-6.931007	-0.259805	-2.165578
46.	1.	0.	-0.178994	2.129889	0.952207
47.	1.	0.	2.162311	2.878329	1.255648
48.	1.	0.	6.656174	2.152651	1.228718
49.	1.	0.	7.969271	0.235030	1.493537
50.	1.	0.	7.845186	-0.549770	-1.448316
51.	1.	0.	8.442815	-1.493858	-0.087291
52.	1.	0.	1.948986	-2.224051	-1.078578
53.	1.	0.	9.099564	1.667912	-0.959119
54.	1.	0.	9.308099	2.161246	0.720812
55.	1.	0.	10.028280	0.643624	0.157572
56.	1.	0.	6.365841	1.765220	-1.531459
57.	1.	0.	6.012400	0.089960	2.528713
58.	1.	0.	4.418284	-0.558700	2.098843
59.	1.	0.	5.573982	-0.614822	-2.520296
60.	1.	0.	5.373056	-2.889952	-0.890231
61.	1.	0.	-3.281561	-1.502266	-2.412089
62.	1.	0.	-0.913110	-0.842941	-2.101393
63.	1.	0.	-6.410802	-0.520994	2.398685
64.	1.	0.	-5.595356	-2.040542	2.054552
65.	1.	0.	-2.420289	0.463578	2.760684
66.	1.	0.	-6.761994	-3.026232	0.260954
67.	1.	0.	-7.528964	-2.063827	-1.015416

68.	1.	0.	-8.973927	-2.513556	0.808397
69.	1.	0.	-4.986563	1.066584	-2.406625
70.	1.	0.	-6.101189	2.410997	-2.660563
71.	1.	0.	-8.507559	1.537577	-2.929084
72.	1.	0.	-8.473191	2.261132	-1.313824
73.	1.	0.	-9.182825	0.657168	-1.547616

**2-4**		Standard Orientation (Ångstroms)			
Center number	Atom number	Type	X	Y	Z
1.	6.	0.	-0.586444	0.662037	0.329016
2.	6.	0.	-1.081517	1.949009	0.592576
3.	6.	0.	-2.438839	2.258772	0.632776
4.	6.	0.	-3.370713	1.253354	0.406358
5.	6.	0.	-2.936414	-0.074270	0.142330
6.	6.	0.	-1.531683	-0.353941	0.094103
7.	8.	0.	-4.679406	1.600642	0.450522
8.	6.	0.	-5.656464	0.673856	-0.026636
9.	6.	0.	-5.363886	-0.798914	0.276483
10.	6.	0.	-3.895341	-1.131301	-0.023635
11.	6.	0.	-7.029207	1.118082	0.532758
12.	6.	0.	-8.166003	0.278703	-0.124408
13.	6.	0.	-7.811897	-1.218414	-0.218516
14.	8.	0.	-3.582054	-2.313399	-0.295629
15.	8.	0.	-1.108454	-1.598155	-0.169552
16.	6.	0.	-9.503288	0.508929	0.585125
17.	8.	0.	-7.052534	1.120933	1.945317
18.	6.	0.	-5.674804	0.609632	-1.579813
19.	8.	0.	-6.127205	-0.707793	-1.923047
20.	6.	0.	-6.376765	-1.449241	-0.706456
21.	8.	0.	-5.587094	-1.171586	1.634954
22.	8.	0.	-6.200715	-2.804675	-0.901021
23.	6.	0.	2.783047	-1.007774	0.995554
24.	6.	0.	3.654678	-0.187654	0.289726
25.	6.	0.	3.165545	0.938673	-0.410623
26.	6.	0.	1.766355	1.213595	-0.405937
27.	6.	0.	0.873043	0.383381	0.304106
28.	6.	0.	1.421244	-0.708613	0.991253
29.	8.	0.	4.977987	-0.512015	0.315563
30.	6.	0.	5.867768	0.060879	-0.668133
31.	6.	0.	5.565057	1.547756	-0.898224

32.	6.	0.	4.090901	1.831804	-1.083989
33.	8.	0.	3.717880	2.819028	-1.739288
34.	8.	0.	1.296013	2.273231	-1.076957
35.	6.	0.	5.691279	-0.758448	-1.962227
36.	8.	0.	6.682506	-0.336870	-2.889814
37.	6.	0.	7.290203	-0.117763	-0.094881
38.	6.	0.	7.679327	-1.569202	0.309045
39.	6.	0.	8.331466	-1.382547	1.687654
40.	6.	0.	7.909962	0.000209	2.153419
41.	8.	0.	7.386714	0.689341	1.095843
42.	8.	0.	8.020913	0.493134	3.243500
43.	6.	0.	8.581420	-2.261416	-0.717032
44.	1.	0.	7.995592	0.285100	-0.829146
45.	1.	0.	6.757829	-2.145161	0.436167
46.	1.	0.	-0.364795	2.744182	0.762003
47.	1.	0.	-2.784730	3.266042	0.831149
48.	1.	0.	-7.165874	2.166439	0.239482
49.	1.	0.	-8.267631	0.644788	-1.153122
50.	1.	0.	-7.934298	-1.709263	0.753154
51.	1.	0.	-8.479776	-1.725358	-0.921315
52.	1.	0.	-1.911249	-2.161102	-0.308840
53.	1.	0.	-9.446208	0.205332	1.633325
54.	1.	0.	-9.782334	1.567396	0.565104
55.	1.	0.	-10.301170	-0.060884	0.098095
56.	1.	0.	-6.727898	0.257190	2.243892
57.	1.	0.	-6.339636	1.368509	-2.005804
58.	1.	0.	-4.669087	0.770731	-1.984219
59.	1.	0.	-5.637057	-2.140377	1.652535
60.	1.	0.	-5.244309	-2.964337	-1.006172
61.	1.	0.	3.171256	-1.866334	1.530556
62.	1.	0.	0.747402	-1.359654	1.536302
63.	1.	0.	5.901326	2.124683	-0.028908
64.	1.	0.	6.112373	1.906930	-1.772681
65.	1.	0.	2.082773	2.719251	-1.495691
66.	1.	0.	4.676813	-0.595127	-2.349329
67.	1.	0.	5.785119	-1.825513	-1.719614
68.	1.	0.	6.451366	-0.675922	-3.763269
69.	1.	0.	8.045373	-2.130339	2.430061
70.	1.	0.	9.427021	-1.388891	1.626705
71.	1.	0.	8.796657	-3.290760	-0.413683
72.	1.	0.	9.536884	-1.732721	-0.810864
73.	1.	0.	8.123683	-2.284426	-1.709635

Table S6. Gibbs free energies[a] and equilibrium populations[b] of low-energy conformers of phomoxanthone N (**3**)

Conformers of **3**	In MeOH	
	ΔG^{a}	P (%) ^{b}
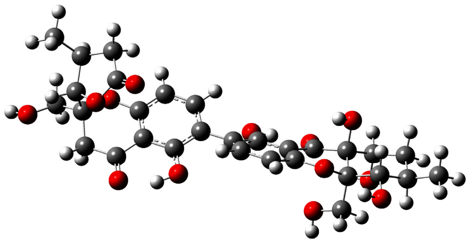 **3-1**	-1342811.87691144	97.88
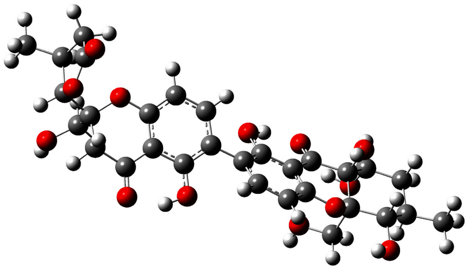 **3-2**	-1342809.50304111	1.77
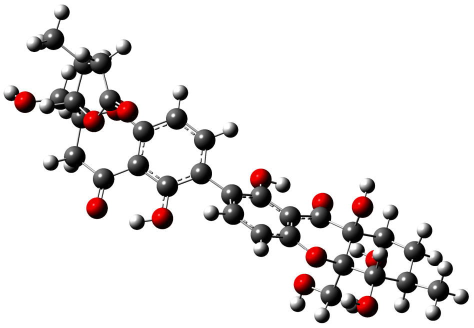 **3-3**	-1342808.5423233	0.35

$^{a}\Delta G$, B3LYP/ 6-31g (d, p), in kcal/mol. ^{b} Boltzmann-population.

Table S7. Cartesian coordinates for the low-energy reoptimized MMFF conformers of phomoxanthone N (**3**) at B3LYP/6-31G(d,p) level of theory in gas

Conformer **3-1**		Standard Orientation (Ångstroms)			
Center Number	Atom	Type	X	Y	Z
1.	6.	0.	0.565724	0.411220	-0.519732
2.	6.	0.	1.076182	1.544075	-1.168848
3.	6.	0.	2.440890	1.806387	-1.297138
4.	6.	0.	3.352767	0.900896	-0.773596
5.	6.	0.	2.898641	-0.280501	-0.140435

6.	6.	0.	1.503537	-0.499311	0.016481
7.	8.	0.	4.687421	1.178719	-0.933378
8.	6.	0.	5.659299	0.473273	-0.091744
9.	6.	0.	5.296101	-1.025972	-0.127207
10.	6.	0.	3.865394	-1.228524	0.386320
11.	6.	0.	7.024749	0.680160	-0.789304
12.	6.	0.	8.137905	-0.149589	-0.122410
13.	6.	0.	7.735579	-1.636712	-0.021179
14.	6.	0.	6.348871	-1.908005	0.571383
15.	8.	0.	3.567773	-2.205543	1.092818
16.	8.	0.	1.071138	-1.588462	0.673788
17.	8.	0.	6.375539	-1.683838	1.974763
18.	6.	0.	9.461776	0.009307	-0.880941
19.	8.	0.	7.404988	2.051753	-0.769549
20.	6.	0.	5.677517	1.123734	1.298500
21.	8.	0.	4.471348	0.839140	1.998249
22.	8.	0.	5.274856	-1.498749	-1.484963
23.	6.	0.	-2.827046	-1.344324	-0.534050
24.	6.	0.	-3.685665	-0.317809	-0.164797
25.	6.	0.	-3.181437	0.978653	0.085610
26.	6.	0.	-1.779484	1.218465	-0.020139
27.	6.	0.	-0.896791	0.180511	-0.393402
28.	6.	0.	-1.461985	-1.077726	-0.643691
29.	8.	0.	-5.015932	-0.613614	-0.068701
30.	6.	0.	-5.888022	0.284509	0.656253
31.	6.	0.	-5.571620	1.749352	0.327866
32.	6.	0.	-4.094475	2.063536	0.398324
33.	8.	0.	-3.707919	3.215051	0.659709
34.	8.	0.	-1.298800	2.442851	0.231794
35.	6.	0.	-5.697200	-0.003309	2.160920
36.	8.	0.	-6.645347	0.776436	2.876666
37.	6.	0.	-7.325181	-0.076025	0.224839
38.	6.	0.	-7.675542	-1.591573	0.233468
39.	6.	0.	-7.515015	-1.982099	-1.243497
40.	6.	0.	-7.626279	-0.674178	-2.008960
41.	8.	0.	-7.554675	0.372091	-1.126174
42.	8.	0.	-7.760383	-0.499622	-3.189922
43.	6.	0.	-9.096364	-1.828642	0.759834
44.	1.	0.	-8.001619	0.492667	0.867170
45.	1.	0.	0.372230	2.261999	-1.572308
46.	1.	0.	2.797914	2.707144	-1.782584
47.	1.	0.	6.909430	0.335407	-1.826314
48.	1.	0.	8.285128	0.247143	0.889168

49.	1.	0.	7.756093	-2.077829	-1.023220
50.	1.	0.	8.473253	-2.173423	0.584421
51.	1.	0.	6.070307	-2.948359	0.358419
52.	1.	0.	1.872143	-2.059578	1.017059
53.	1.	0.	5.507333	-1.960904	2.304960
54.	1.	0.	9.376204	-0.378591	-1.903203
55.	1.	0.	10.261960	-0.545342	-0.380219
56.	1.	0.	9.753194	1.060082	-0.939534
57.	1.	0.	6.720671	2.539761	-1.248535
58.	1.	0.	5.806720	2.201995	1.148575
59.	1.	0.	6.538840	0.737817	1.847601
60.	1.	0.	4.587448	1.121329	2.913747
61.	1.	0.	4.796593	-0.851181	-2.022951
62.	1.	0.	-3.224197	-2.334904	-0.722199
63.	1.	0.	-0.798767	-1.888405	-0.921498
64.	1.	0.	-6.111486	2.409405	1.010033
65.	1.	0.	-5.908178	1.971561	-0.691350
66.	1.	0.	-2.080594	3.014576	0.467779
67.	1.	0.	-4.665869	0.249123	2.441587
68.	1.	0.	-5.833405	-1.078251	2.342097
69.	1.	0.	-6.412120	0.761777	3.812837
70.	1.	0.	-6.965777	-2.143148	0.854201
71.	1.	0.	-6.529899	-2.407533	-1.455137
72.	1.	0.	-8.269828	-2.685299	-1.604089
73.	1.	0.	-9.355547	-2.891248	0.718631
74.	1.	0.	-9.834119	-1.280142	0.163163
75.	1.	0.	-9.191054	-1.496013	1.798538

Conformer **3-2**		Standard Orientation (Ångstroms)			
Center Number	Atom	Type	X	Y	Z
1.	6.	0.	0.352273	0.191645	0.093632
2.	6.	0.	0.730139	1.472471	0.526003
3.	6.	0.	2.056414	1.885937	0.639821
4.	6.	0.	3.073995	1.003035	0.299752
5.	6.	0.	2.752193	-0.290037	-0.180277
6.	6.	0.	1.391084	-0.698473	-0.249477
7.	8.	0.	4.355637	1.436858	0.447091
8.	6.	0.	5.450596	0.481751	0.357144
9.	6.	0.	5.172280	-0.497209	-0.798033
10.	6.	0.	3.813296	-1.176250	-0.598176
11.	6.	0.	6.702443	1.323573	0.011176

12.	6.	0.	7.937510	0.436390	-0.234771
13.	6.	0.	7.637617	-0.665562	-1.274002
14.	6.	0.	6.349771	-1.465765	-1.041593
15.	8.	0.	3.653456	-2.366534	-0.931025
16.	8.	0.	1.092284	-1.944944	-0.659705
17.	8.	0.	6.542386	-2.380217	0.026637
18.	6.	0.	9.137812	1.286032	-0.671617
19.	8.	0.	7.003788	2.233665	1.064521
20.	6.	0.	5.622042	-0.173272	1.737895
21.	8.	0.	4.532397	-1.051183	2.001472
22.	8.	0.	5.009692	0.315201	-1.977206
23.	6.	0.	-2.911700	-1.271926	-1.248239
24.	6.	0.	-3.806369	-0.956023	-0.235611
25.	6.	0.	-3.364010	-0.280725	0.921236
26.	6.	0.	-1.997198	0.104941	1.028986
27.	6.	0.	-1.076485	-0.206296	0.002508
28.	6.	0.	-1.574201	-0.897655	-1.109709
29.	8.	0.	-5.105542	-1.350167	-0.413023
30.	6.	0.	-6.158006	-0.841864	0.433706
31.	6.	0.	-5.673012	-0.631118	1.877923
32.	6.	0.	-4.294144	-0.012850	1.999233
33.	8.	0.	-3.982768	0.602051	3.030633
34.	8.	0.	-1.578226	0.754531	2.123216
35.	6.	0.	-7.206236	-1.964633	0.373715
36.	8.	0.	-8.360122	-1.534323	1.081420
37.	6.	0.	-6.734927	0.465680	-0.161709
38.	6.	0.	-7.127884	0.420139	-1.664223
39.	6.	0.	-6.605732	1.763665	-2.196180
40.	6.	0.	-5.603057	2.247887	-1.162828
41.	8.	0.	-5.736616	1.498863	-0.025722
42.	8.	0.	-4.813408	3.149096	-1.245185
43.	6.	0.	-8.626791	0.207596	-1.899182
44.	1.	0.	-7.594884	0.751777	0.454295
45.	1.	0.	-0.053258	2.167761	0.803080
46.	1.	0.	2.307366	2.875994	1.002057
47.	1.	0.	6.467469	1.873317	-0.909276
48.	1.	0.	8.197610	-0.038617	0.718414
49.	1.	0.	7.563516	-0.200613	-2.262902
50.	1.	0.	8.473677	-1.371671	-1.311333
51.	1.	0.	6.111584	-2.022646	-1.962156
52.	1.	0.	1.948454	-2.417253	-0.809537
53.	1.	0.	5.713938	-2.875441	0.114452
54.	1.	0.	8.938046	1.783102	-1.628568

55.	1.	0.	10.028670	0.662203	-0.798562
56.	1.	0.	9.356715	2.055894	0.071188
57.	1.	0.	6.239153	2.820564	1.148288
58.	1.	0.	5.664932	0.642965	2.467802
59.	1.	0.	6.566673	-0.720801	1.762069
60.	1.	0.	4.678983	-1.455308	2.865575
61.	1.	0.	4.785666	-0.280103	-2.707169
62.	1.	0.	-3.263243	-1.791839	-2.131823
63.	1.	0.	-0.882633	-1.149995	-1.904648
64.	1.	0.	-5.611826	-1.606760	2.378842
65.	1.	0.	-6.386380	-0.030948	2.445342
66.	1.	0.	-2.373531	0.871332	2.710016
67.	1.	0.	-6.759424	-2.863817	0.819315
68.	1.	0.	-7.427792	-2.191442	-0.678163
69.	1.	0.	-8.973267	-2.276654	1.146529
70.	1.	0.	-6.560129	-0.388924	-2.135179
71.	1.	0.	-6.132982	1.711313	-3.179047
72.	1.	0.	-7.402806	2.515126	-2.259254
73.	1.	0.	-8.846010	0.136780	-2.969152
74.	1.	0.	-9.203866	1.046265	-1.493440
75.	1.	0.	-8.992371	-0.701225	-1.414654

Conformer **3-3**		Standard Orientation (Ångstroms)			
Center Number	Atom	Type	X	Y	Z
1.	6.	0.	0.292380	0.158100	0.088925
2.	6.	0.	0.654963	1.451337	0.491849
3.	6.	0.	1.978682	1.882705	0.589648
4.	6.	0.	3.000327	1.004707	0.256914
5.	6.	0.	2.695409	-0.302244	-0.193237
6.	6.	0.	1.341157	-0.730726	-0.235903
7.	8.	0.	4.289532	1.468441	0.342219
8.	6.	0.	5.389940	0.499980	0.347978
9.	6.	0.	5.123521	-0.510049	-0.787697
10.	6.	0.	3.773470	-1.201772	-0.564148
11.	6.	0.	6.655341	1.322584	0.007418
12.	6.	0.	7.885855	0.418360	-0.194372
13.	6.	0.	7.594823	-0.688883	-1.229865
14.	6.	0.	6.300706	-1.478809	-1.008554
15.	8.	0.	3.620661	-2.409321	-0.811676

16.	8.	0.	1.055329	-1.992078	-0.601509
17.	8.	0.	6.463532	-2.368204	0.088188
18.	6.	0.	9.106905	1.248952	-0.609616
19.	8.	0.	6.944216	2.264209	1.035208
20.	6.	0.	5.516684	-0.095515	1.757290
21.	8.	0.	4.401799	-0.931687	2.045299
22.	8.	0.	4.987505	0.178315	-2.042895
23.	6.	0.	-2.950879	-1.373490	-1.222807
24.	6.	0.	-3.856480	-1.025592	-0.229139
25.	6.	0.	-3.426242	-0.312662	0.910186
26.	6.	0.	-2.060784	0.078490	1.018178
27.	6.	0.	-1.131666	-0.257471	0.006268
28.	6.	0.	-1.615865	-0.988911	-1.085879
29.	8.	0.	-5.155546	-1.415531	-0.409002
30.	6.	0.	-6.209596	-0.844177	0.400003
31.	6.	0.	-5.748687	-0.625489	1.849347
32.	6.	0.	-4.365552	-0.020837	1.977519
33.	8.	0.	-4.053967	0.606828	3.000261
34.	8.	0.	-1.651904	0.757755	2.096521
35.	6.	0.	-7.306028	-1.922011	0.342027
36.	8.	0.	-8.413237	-1.463280	1.102463
37.	6.	0.	-6.741879	0.462266	-0.234493
38.	6.	0.	-6.904995	0.446126	-1.782189
39.	6.	0.	-5.694475	1.261846	-2.261652
40.	6.	0.	-5.257760	2.072073	-1.052347
41.	8.	0.	-5.866121	1.568383	0.068170
42.	8.	0.	-4.501260	3.003373	-1.000701
43.	6.	0.	-8.242641	1.062772	-2.208982
44.	1.	0.	-7.693528	0.675871	0.260208
45.	1.	0.	-0.135048	2.143192	0.758095
46.	1.	0.	2.220392	2.882751	0.930232
47.	1.	0.	6.450717	1.846378	-0.936726
48.	1.	0.	8.114954	-0.049774	0.770322
49.	1.	0.	7.535685	-0.235232	-2.224730
50.	1.	0.	8.427178	-1.400191	-1.246505
51.	1.	0.	6.069134	-2.041705	-1.922241
52.	1.	0.	1.917391	-2.462754	-0.727702
53.	1.	0.	5.655448	-2.902901	0.118825
54.	1.	0.	8.936234	1.739157	-1.575705
55.	1.	0.	9.990810	0.610981	-0.711182
56.	1.	0.	9.320790	2.022573	0.130748
57.	1.	0.	6.177474	2.850625	1.100334
58.	1.	0.	5.576133	0.746178	2.457204

59.	1.	0.	6.446816	-0.665778	1.810050
60.	1.	0.	4.578593	-1.383765	2.879371
61.	1.	0.	4.395037	0.932733	-1.911757
62.	1.	0.	-3.291945	-1.929964	-2.088192
63.	1.	0.	-0.916770	-1.264956	-1.866157
64.	1.	0.	-5.708209	-1.597333	2.359661
65.	1.	0.	-6.462258	-0.009040	2.397931
66.	1.	0.	-2.450599	0.880565	2.679016
67.	1.	0.	-6.879888	-2.850815	0.745931
68.	1.	0.	-7.578105	-2.110665	-0.706322
69.	1.	0.	-9.080887	-2.159637	1.120959
70.	1.	0.	-6.843462	-0.579540	-2.152739
71.	1.	0.	-4.850456	0.628158	-2.552931
72.	1.	0.	-5.909875	1.931185	-3.097907
73.	1.	0.	-8.334920	1.080017	-3.299545
74.	1.	0.	-8.334861	2.093709	-1.848475
75.	1.	0.	-9.086102	0.492369	-1.806469
